# Shape‐Selective Ultramicroporous Carbon Membranes for Sub‐0.1 nm Organic Liquid Separation

**DOI:** 10.1002/advs.202004999

**Published:** 2021-07-11

**Authors:** Hyeokjun Seo, Sunghyun Yoon, Banseok Oh, Yongchul G. Chung, Dong‐Yeun Koh

**Affiliations:** ^1^ Department of Chemical and Biomolecular Engineering (BK‐21 Plus) Korea Advanced Institute of Science and Technology Daejeon 34141 South Korea; ^2^ School of Chemical Engineering Pusan National University Busan 46241 South Korea

**Keywords:** nanoporous carbon, organic solvent forward osmosis (OSFO), pore rigidity, shape selectivity, solvent‐solvent separation

## Abstract

Liquid‐phase chemical separations from complex mixtures of hydrocarbon molecules into singular components are large‐scale and energy‐intensive processes. Membranes with molecular specificity that efficiently separate molecules of similar size and shape can avoid phase changes, thereby reducing the energy intensity of the process. Here, forward osmosis molecular differentiation of hexane isomers through a combination of size‐ and shape‐based separation of molecules is demonstrated. An ultramicroporous carbon membrane produced with 6FDA‐polyimides realized the separation of isomers for different shapes of di‐branched, mono‐branched, and linear molecules. The draw solvents provide the driving force for fractionation of hexane isomers with a sub‐0.1 nm size difference at room temperature without liquid‐phase pressurization. Such membranes could perform bulk chemical separations of organic liquids to achieve major reductions in the energy intensity of the separation processes.

## Graphical abstract

The nanoporous carbon derived from 6FDA‐based polyimide reveals confined pore structure sized suitable for the separation of liquid hydrocarbons. Rigid ultramicropores in 6FDA−DAM carbon molecular sieve (CMS) hollow fiber membranes enable molecular discrimination of hexane isomers based on size and shape difference via ‘organic solvent forward osmosis (OSFO)’ without pressurization at room temperature.

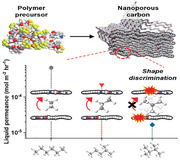

## 1 Introduction

Advanced separation technology that can differentiate mixtures of molecules based on their size and shape differences at the molecular level is an enabling technology towards a more energy‐efficient society. Currently, separation processes are responsible for 10–15% of worldwide energy consumption since the molecular selectivity of the process was achieved by thermal properties (e.g., boiling points) rather than the size and shape of the molecule.^[^ ^1^ ^]^ Molecularly‐selective membrane processes that can fractionate hydrocarbon mixtures based on the molecular size and shape are next‐generation disruptive technologies that can provide a 10‐fold increase in energy efficiency over thermally‐driven separation processes.^[^ ^2^ ^]^ By benchmarking the success of membranes in the field of seawater desalination and gas separations in the last few decades, membrane‐based separation processes have evolved to provide low‐energy solutions to organic liquid separations.^[^ ^3^, ^4^, ^5^, ^6^, ^7^ ^]^ The efficient purification of high‐value alkane isomers (pentanes and hexanes), methylated aromatics (xylenes, benzene, toluene) is especially challenging because molecules are chemically inert and have similar boiling points. Organic solvent reverse osmosis (OSRO) has recently emerged as a highly‐efficient separation process capable of size‐selective separation of liquid aromatics, including xylene isomers, aliphatic compounds, and alcohols.^[^ ^8^, ^9^, ^10^, ^11^, ^12^, ^13^, ^14^, ^15^, ^16^ ^]^ However, OSRO requires excessive liquid pressure in the feed stream to overcome the transmembrane osmotic pressure gradient. A realistic feed with complex mixtures is often challenging for OSRO when the light component in the feed is dilute that drives up the osmotic pressure over hundreds of bars.^[^ ^7^ ^]^ To resolve this, we explore the potential for organic solvent forward osmosis (OSFO) that does not require excessive transmembrane pressure to separate hexane isomers via size‐ and shape‐selective carbon molecular sieve (CMS) hollow fiber membranes.

The worldwide production of hexanes and pentanes has reached an enormous scale of up to two million barrels per year.^[^ ^17^ ^]^ The large‐scale separation is still practiced by energy‐intensive cryogenic distillation, although some adsorptive separation technology such as the ISOSIV process has been developed.^[^ ^18^ ^]^ The catalytic isomerization before the fractionation produces complex mixtures of hexane isomers composed of 10–25% of each of the five different isomers.^[^ ^18^ ^]^ The research octane number (RON) of the mixture depends on the molecular size and shape of the hexane isomers, with di‐branched isomers having the highest ratings and the linear isomer having the lowest. Owing to their shape differences, linear, mono‐branched, and di‐branched hexane isomers possess kinetic diameters that differ by about 0.1 nm. Ultramicropores designed with suitable pore size and curvature would enable the kinetic separation of such complex mixtures.

Numerous solid adsorbents, including zeolites, silicas, and metal–organic frameworks can sieve hexane isomers to achieve high RON mixtures.^[^ ^19^, ^20^, ^21^, ^22^, ^23^, ^24^, ^25^, ^26^, ^27^, ^28^ ^]^ However, many of these materials can only discriminate linear hexane from mono‐ and di‐branched hexanes, demanding additional fractionation of di‐branched hexanes from mono‐branched hexanes.^[^ ^21^, ^22^, ^23^, ^24^, ^25^, ^26^ ^]^ For instance, a beta zeolite membrane enables the separation of linear *n*‐hexane from the mono‐branched (3‐methylpentane) and di‐branched isomer (2,2‐dimethylbutane and 2,3‐dimethylbutane); however, the separation capability of the material was tested at a very low‐pressure regime, which does not reflect the practical conditions.^[^ ^20^ ^]^ Several recent studies suggested that the metal–organic framework with a triangular pore/window^[^ ^27^, ^28^ ^]^ could achieve the shape‐selective separation of the di‐branched hexane isomer from the mixture of mono‐branched and linear isomers via the sorption process at practical pressure ranges. Therefore, a significant opportunity is present for dramatic reductions in energy and carbon intensity with advanced separations on microporous materials that can “sieve” molecules without bulk phase transitions.

The microporous carbon membranes could be potential game‐changers in organic solvent separation due to their molecular selectivity and pore tunability in addition to excellent solvent resistance. A recent report has suggested that graphene oxide membranes with a pore tuning approach, including size modification of GO sheets, could be applied to OSFO process with excellent selectivity in addition to high permeability.^[^ ^29^ ^]^ Similarly, the carbon molecular sieve (CMS) with a hierarchical and rigid micropore structure can be used as an advanced membrane material for the organic solvent forward osmosis (OSFO) process. CMS provides the best combination of the ultramicroporosity with size‐ and shape‐selectivity, solvent‐resistance, and importantly, scalability in membrane fabrication.^[^ ^30^, ^31^ ^]^ In the forward osmosis process, the transmembrane osmotic pressure gradient is modulated by the draw solution, which extracts the upstream target molecule downstream. In bulk chemical separations, the same strategy can be used to achieve organic solvent separations. OSFO has been conceptualized in various studies^[^ ^7^, ^31^, ^32^ ^]^ and utilized, to somewhat extent, in organic solvent separations for small pharmaceutical molecules.^[^ ^33^, ^34^ ^]^ The membrane molecular weight cut‐off for the previous studies was reported to be greater than 400 g mol^−1^,^[^ ^33^, ^34^ ^]^ which represents “solute”‐“solvent” separations.^[^ ^6^ ^]^ For the demonstration of OSFO in complex hydrocarbon separations, molecular specificity in the reverse osmosis range (< 100 g mol^−1^) is necessary^[^ ^35^ ^]^ to promote “solvent”‐“solvent” separations (Note S1, Figure S1, and Table S1, Supporting Information).

**Figure 1:**
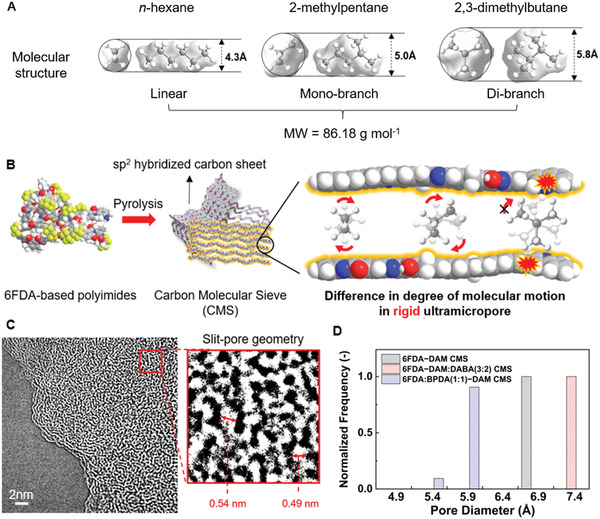
A) Representative hexane isomers for each branch state (shape): *n‐*hexane, linear/2‐methylpentane, mono‐branch/2,3‐dimethylbutane, di‐branch. B) Transformation of 6FDA‐based polyimides into CMS with rigid ultramicroporous structure enabling shape discrimination. C) TEM image of CMS membrane visualizing slit‐pore geometry. D) Estimated pore size distribution of three different CMS membranes calculated from 87 K Ar physisorption.

In this work, we utilize CMS hollow fiber membranes to demonstrate size‐ and shape‐selective OSFO separation of hexane isomers in the liquid phase (**Figure** **1**A,B). The OSFO process design is complete only with a judicious selection of the draw solvent that is large enough to minimize the back diffusion while keeping the transmembrane chemical potential gradient as a driving force for separation. Three different hexane isomers were selected (*n*‐hexane/*n*‐hex, linear; 2‐methylpentane/2‐MP, mono‐branched; 2,3‐dimethylbutane/2,3‐DMB, di‐branched), and two different draw solvents (1,3,5‐trimethylbenzene and 1,3,5‐triisopropylbenzene) were tested in an OSFO setup (Figure S2, Supporting Information). Our work demonstrates that the diffusive discrimination of hexane isomers of CMS membrane was successfully translated into the steady‐state liquid‐phase separation process. The CMS membranes from three‐class of 6FDA‐polyimides have been proven to show significant diffusion selectivities of linear or mono‐branched isomer over di‐branched isomer (up to 70) regardless of their difference in chain flexibility, where the molecular sieving based on size‐ or shape‐selectivity was varied with chain rigidity. The CMS hollow fiber membrane based on 6FDA−DAM displayed significant shape selectivity (i.e., entropic selectivity) of 2‐MP over 2,3‐DMB, successfully fractionating high‐value di‐branched isomer from mixture without any hydraulic pressurization and phase change.

## 2 Results and Discussion

For the sharp discrimination of alkanes, the ultramicropores within CMS need to be similarly sized with the target molecules, and a rigid and confined structure needs to be provided for shape selectivity. Various parameters, including polymeric precursor,^[^ ^36^, ^37^ ^]^ pyrolysis conditions,^[^ ^38^, ^39^, ^40^ ^]^ and pre‐/post‐treatment,^[^ ^41^, ^42^, ^43^, ^44^, ^45^ ^]^ enable the fine tuning of pore size and structure of CMS. For example, higher pyrolysis temperature leads to tighter structure or smaller ultramicropore size. Several methods for selective tuning of ultramicropores to match their size to the penetrant molecules have been reported to enhance the selectivity of the membrane. The oxygen doping on carbon structure causes selective sorption of oxygen molecules on the ultramicropores rather than micropores, resulting in higher selectivity.^[^ ^39^, ^40^ ^]^ Meanwhile, post‐pyrolysis heat‐treatment process called “hyperaging” facilitates tightening and widening of ultramicropores when the CMS membranes are treated at below 250 and above 250 °C, respectively.^45^ In this study, we sought to match the pore size distribution of carbon molecular sieve membranes to the size of hexane isomers by choosing the fluorinated polyimides as polymeric precursors followed by pyrolysis at 500 °C. Carbon molecular sieve membranes derived from 6FDA (4,4’‐(hexafluoroisopropylidene)diphthalic anhydride) based polyimides were reported to show higher permeability for several light hydrocarbon gas molecules compared to that of the other precursors such as commercial polyimides (Matrimid®, Kapton, Torlon, P84), polybenzimidazole (PBI), and polymers with intrinsic microporosity (PIMs).^[^ ^46^, ^47^, ^48^, ^49^, ^50^, ^51^, ^52^, ^53^, ^54^, ^55^ ^]^ The inefficient chain packing originating from the bulky CF_3_ groups in 6FDA‐based polyimides allowed the formation of highly permeable CMS membranes while its molecular specificity was retained, which is different from other “tight” CMS membranes with most of the micropores smaller than 4 Å.^[^ ^43^, ^44^ ^]^ We prepared three different polyimides (Figure S3, Supporting Information; 6FDA−DAM, 6FDA−DAM:DABA(3:2), and 6FDA:BPDA(1:1)−DAM) with different chain flexibilities, which impacts the final CMS structure when pyrolyzed. We further employed lower pyrolysis temperature than other CMSs in gas separation (i.e., *T*_p_ > 550 °C) to ensure the similarity of the size between ultramicropore and hexane isomers. The molecular weight and polydispersity index (PDI) of the polyimides used in this study are shown in Table S2 (Supporting Information).

Carbon molecular sieve membranes were fabricated via the pyrolysis of these precursors at 500 °C under low oxygen concentration (< 30 ppm). Thermal degradation reorganizes the polymer backbone via aromatization, where these sp^2^‐hybridized carbon sheets undergo random stacking with entropically‐driven volume exclusion (**Figure** 1B). Elemental analysis of the three‐class of 6FDA polyimides and the corresponding CMS membranes showed a compositional change into the carbon‐enriched structure after pyrolysis (Table S3, Supporting Information). Fourier‐transform infrared (FT‐IR) spectra (Figure S4, Supporting Information) of the CMS membranes clearly show that the bond cleavage occurred in the polymer backbone via the reduction in peak intensities of several functional groups (e.g., C—N stretching, C═O bending). All three CMS membranes showed weak C═O stretching bands (1740 and 1770 cm^−1^) derived from the imide linkage. Thermogravimetric analysis (TGA) also indicates that the decomposition of the 6FDA polyimides starts at 450 °C (Figure S5, Supporting Information); therefore, the CMS membranes prepared by the pyrolysis at 500 °C could lead to an overall rigid microporous structure while still maintaining a small portion of the polymeric properties (i.e., chain flexibility). High‐Resolution Transmission electron microscopy (HR‐TEM) images (**Figure** 1C) reveals slit‐pore geometry in CMS and pore size distribution similar with kinetic diameter of hexane isomers. X‐ray diffraction (XRD) and Raman spectra (Figures S6 and S7, Supporting Information) further confirmed the formation of a turbostratic graphitic network in all of the CMS membranes.

The pore structure of microporous materials could be studied based on several sorption‐based methods and permporometry.^[^ ^56^, ^57^, ^58^ ^]^ CMS membranes in this study were analyzed via physisorption of three different adsorbates: N_2_, CO_2,_ and Ar. N_2_ physisorption at 77K (Figure S8, Supporting Information) showed Type 1 isotherms for all CMS membranes, indicating a microporous structure with ultramicropores. As shown in Figure S8 (Supporting Information), the Brunauer–Emmett–Teller (BET) surface areas of the CMS derived from 6FDA−DAM and 6FDA−DAM:DABA are similar; on the other hand, the CMS derived from 6FDA:BPDA−DAM has both a lower BET surface area and lower pore volume—implying the difference in micropore openness among CMS derived from different precursors. A similar structural difference was also detected in the CO_2_ isotherm measured at 273 K (Figure S9, Supporting Information); however, in the case of CO_2_ sorption, the isotherms for 6FDA−DAM and 6FDA−DAM:DABA showed different uptake patterns for affinity toward sorbate, again suggesting the structural difference in each CMS. We have further employed argon physisorption at 87K in three different CMSs to clarify difference of pore structure between the CMSs (Figure S10, Supporting Information). Monoatomic argon with no quadrupole moment is relatively free from interactions with delocalized electrons on sp^2^‐hybridized carbon, is a suitable probe molecule to show the realistic pore structure of the CMS membranes. Comparing the 87 K argon physisorption data with 77 K N_2_ and 273 K CO_2_ physisorption results, the micropore openness increase in the order of CMS derived from 6FDA:BPDA–DAM, 6FDA–DAM and 6FDA–DAM:DABA. We generated graphite‐sheet models for each CMS membrane based on the physisorption data with three different adsorbates (Note S2, Figures S11 and S12, Supporting Information). The pore size distribution of the CMS models was estimated based on the atomistic molecular simulation combined with linear programming (see the Supporting Information for details of the molecular simulation). **Figure** 1D shows the estimated pore size distribution of the graphite model based on parallel carbon sheets calculated from argon physisorption data. Molecular simulation results show that the 6FDA−DAM CMS and 6FDA−DAM:DABA CMS possess a higher average pore size (6.9 and 7.4 Å), which is larger than the kinetic diameter of di‐branched 2,3‐DMB (5.8 Å). However, we found that the 6FDA:BPDA‐DAM CMS has the smallest average pore size that can fractionate di‐branched isomer from mono‐ and linear hexane isomers. Therefore, we precisely measured the diffusion of hexane isomers in these CMS membranes.

To further probe the size‐ and shape‐selectivity associated with these CMS, we conducted gravimetric vapor sorption experiments at 25, 35, and 45 °C with the flat sheet CMS membranes with the thickness of ≈10 µm. As illustrated in Figure S13 (Supporting Information), the prepared CMS membranes have homogeneous surfaces, represented by nanoscale surface roughness. Thus, we have utilized the flat sheet CMS membranes with uniform surface barrieres for the diffusional analysis (Figures S14–S16, Supporting Information). The rate at hexane isomer diffusion in different CMS membranes was expected to vary due to the different textural properties of all three CMS membrane candidates. Based on the isotherms generated with three adsorbates, *n*‐hex, 2‐MP, and 2,3‐DMB, the equilibrium sorption coefficients were calculated. The sorption coefficients (S_i_) increased in the order of 6FDA:BPDA−DAM, 6FDA−DAM, and 6FDA−DAM:DABA derived CMS, where the *n*‐hex sorption was highest for all the tested membranes. These sorption coefficients correlate with the pore volume measured by 77 K N_2_ physisorption. All the CMS membranes exhibited high uptakes of hexane isomers but had only moderately lower sorption selectivity (S_i_/S_j_ < 2.5, Table S4, Supporting Information).

**Figure 2:**
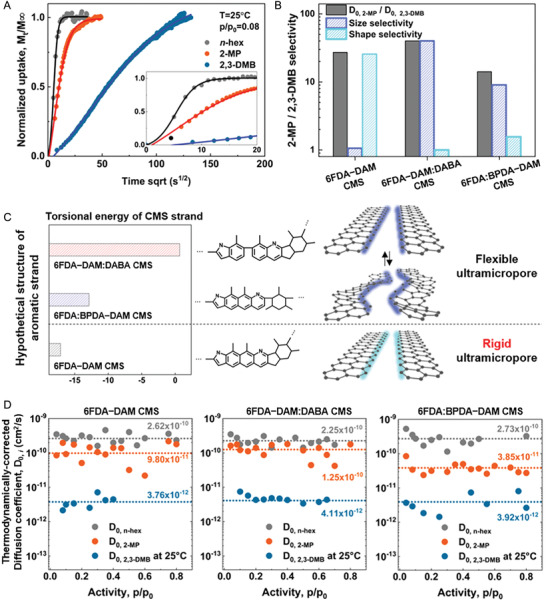
A) Fickian model fitting for *n*‐hex, 2‐MP, and 2,3‐DMB in 6FDA−DAM CMS membranes. B) Contribution of size (enthalpic) selectivity and shape (entropic) selectivity on diffusion selectivity for 2‐MP/2,3‐DMB in each CMS membrane, C) Hypothetical structure of aromatic strands derived from three different 6FDA‐based polyimides, showing different torsional freedom of strands and corresponding ultramicropore. D) Thermodynamically‐corrected diffusion coefficients versus activity in three different CMS membranes and averaged diffusion coefficients of each isomer in each CMS membrane.

The rigid pore structures in the CMS membranes exhibited high diffusion selectivity, including the shape selectivity (**Figure** **2**A–D). Uptake curves of hexane isomers for 6FDA−DAM CMS showed significantly delayed kinetics for branched isomers, especially for 2,3‐DMB (**Figure** 2A). Thermodynamically‐corrected (i.e., Darken‐corrected) diffusion coefficients of hexane isomers for the three different CMS membranes at 25, 35, and 45 °C are shown in **Figure** 2D; and Figures S17–S19 (Supporting Information). Since the penetrants did not strongly sorb onto the CMS micropores, the thermodynamically‐corrected diffusivities were independent of the penetrant concentration. The diffusion coefficients of the hexane isomer for all the membranes increases as the kinetic diameter of the isomer decreases and the diffusion coefficients were in the order of *n*‐hex, 2‐MP, and 2,3‐DMB (**Figure** 2D). The diffusion data are supported by the structural analyses of the model graphite structures showing the ultramicropore aperture in the 6FDA:BPDA−DAM derived CMS was the most compact compared to other membranes. As depicted in Table S5 (Supporting Information) and **Figure** 2D, all the CMS membranes showed low‐to‐moderate diffusion selectivity of a linear over mono‐branched isomer. The high diffusion selectivities for linear or mono‐branched isomer over di‐branched isomer (*D*_0,_ *_n_*_‐hex_/*D*_0, 2,3‐DMB_ over 54.9 and *D*_0, 2‐MP_/*D*_0, 2,3‐DMB_ over 9.9) were demonstrated with all three CMS membranes tested. The results again suggest that selective removal of linear and mono‐branched isomers is achievable through any of these CMS membranes from the complex mixture of hexane isomers.

Shape‐selectivity (i.e., entropic selectivity) is the molecular discrimination mechanism for microporous materials showing a rigid and confined pore structure, restricting the penetrant's shape and rotational/vibrational degrees of freedom (**Figure** 1B).^[^ ^59^ ^]^ To further elucidate the shape‐selectivity in those CMS membranes, diffusion analysis via transition state theory (see Note S3, Supporting Information) was performed. As shown in Figure S20 (Supporting Information), the average diffusion coefficients for all cases increased with the temperature, reflecting the positive activation energy of the diffusion process. In all CMSs we tested, the larger penetrants (e.g., 2,3‐DMB) demanded higher activation energy for the localization to transition state as the diffusion initiated. We estimated both the enthalpic (based on kinetic diameter difference) and entropic (based on shape difference) contribution on diffusion selectivity using Equation (S8) (Supporting Information) and the calculated values are tabulated in Table S5 (Supporting Information). Interestingly, the CMS membrane derived from 6FDA−DAM showed an apparent entropic selectivity for the 2‐MP/2,3‐DMB pair, as depicted in **Figure** 2B; and Table S5 (Supporting Information). Except for this pair, all CMS membranes showed only size‐based discrimination (i.e., enthalpic selectivity) of hexane isomers. It should be pointed out that the diffusion selectivity of 2‐MP/2,3‐DMB for 6FDA−DAM derived CMS was almost exclusively achieved by the shape‐based (entropic) selection process. As discussed in the previous section, all three CMS membranes were prepared at a pyrolysis temperature of 500 °C, which is slightly higher than the polymeric degradation temperature of the precursors, might resulting in the flexibility of aromatic strands composing ultramicropores. Even though some degree of flexibility remained, the micropore walls in 6FDA−DAM derived CMS were rigid and confined enough to induce shape selectivity between mono‐branched and di‐branched hexane isomers (**Figures** **1**B  

and 2C). As shown in **Table** **1**, within the micropores of 6FDA−DAM CMS, adsorbed *n*‐hexane can diffuse through the pores with lower activation energy (6.64 kJ mol^−1^) compared to those of larger isomers of 2‐MP and 2,3‐DMB (27.75 and 27.90 kJ mol^−1^, respectively).

**Table 1:** Estimated activation energy of diffusion of each isomer in each CMS membrane

Sample	Activation energy of diffusion, *E*_d, i_ [kJ mol^−1^]		
	*E*_d,_ *_n‐_*_hex_	*E*_d, 2‐MP_	*E*_d, 2,3‐DMB_
6FDA−DAM CMS	6.64	27.75	27.90
6FDA−DAM:DABA(3:2) CMS	6.18	19.60	36.45
6FDA:BPDA(1:1)−DAM CMS	2.01	40.69	46.32

The shape selectivity of isomers can be realized when the molecular motions, including the rotational and vibrational modes during the translational diffusion process, were restricted in the confined micropores. Based on the textural data, all the 6FDA based CMS might possess confined “slit‐like” ultramicropores composed of aromatic strands. However, the pore rigidity and pore confinement are essential features that provide CMS a shape selectivity. Due to the different physical properties of three 6FDA based polyimides, the polymeric precursors undergo different thermal degradation or decomposition rate. Thus, three CMS membranes possess different pore structures when pyrolyzed at the same conditions, as supported by the physisorption experiments and diffusion analysis. Based on the diffusion analysis, the aromatic strands and the ultramicropores within 6FDA–DAM CMS seem to be rigid enough to show shape‐selectivity for 2‐MP/2,3‐DMB pair. Rotating or vibrating 2,3‐DMB cannot diffuse through the ultramicropore, whereas 2‐MP can freely rotate or vibrate during the diffusion process due to the smaller size and different shape. The ultramicropores within 6FDA–DAM:DABA CMS or 6FDA:BPDA–DAM CMS might be composed of flexible aromatic strands (**Figure** 2C), which could exhibit conformational vibration of the polymer chain, leading to lower shape selectivity for 2‐MP and 2,3‐DMB pair (Figure S21, Supporting Information). Significant differences in diffusional activation energies of 2‐MP and 2,3‐DMB in 6FDA:BPDA−DAM CMS and 6FDA−DAM:DABA CMS (Δ*E*_6FDA:BPDA–DAM CMS_ = 5.63 kJ mol^−1^ and Δ*E*_6FDA–DAM:DABA CMS_ = 16.85 kJ mol^−1^) suggest that the molecular size difference between 2‐MP and 2,3‐DMB was sufficient to induce the diffusion selectivity in these CMS membranes. It is interesting to note that both 2‐MP and 2,3‐DMB require similar activation energy of diffusion in 6FDA−DAM CMS, thus, leaving entropic selectivity as a singular contribution to diffusion selectivity. Therefore, only 6FDA−DAM derived CMS demonstrated both size‐ and shape‐selectivity in the diffusion process of hexane isomers. We then carried out solvent separation by synthesizing CMS hollow fiber membranes derived from 6FDA−DAM to demonstrate its capability of both size‐ and shape‐selective separation in organic liquid mixtures.

**Figure 3:**
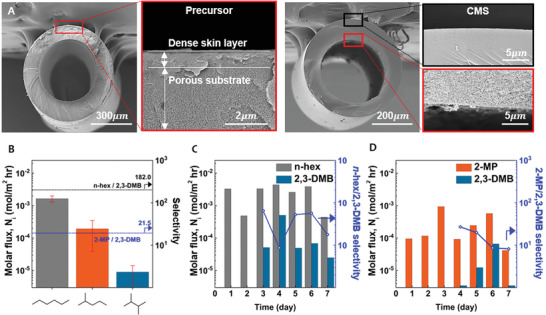
A) Cross‐sectional SEM image of precursor (left) and CMS (right) hollow fiber membrane. B) Single component permeation data. C,D) Binary mixture permeation data versus experimental time.

The dry‐jet/wet‐quench spinning technology (Experimental Section, Figure S22, and Table S6, Supporting Information) was used to fabricate thin‐skinned hollow fiber membranes with an in‐house synthesized 6FDA−DAM precursor, followed by the same pyrolysis protocol performed at 500 °C. The cross‐sectional scanning electron microscopy (SEM) image (**Figure** **3**A) of the precursor asymmetric hollow fiber shows an estimated skin layer thickness of ∼1 µm, where the CMS hollow fiber shows an asymmetric structure with an increased skin layer (up to 30–80 µm depending on the location) due to substructure collapse during the pyrolysis.^[^ ^60^ ^]^ OSFO requires a homogeneous defect‐free membrane structure with reproducible performances. Therefore, the morphological homogeneity of the hollow fiber membranes and gaseous He/N_2_ selectivity were examined before each solvent separation experiment. The SEM images of polymeric precursor and CMS fibers collected from (i) to (iv) in Figure S23 (Supporting Information) show the uniform, homogeneous morphology is maintained along the longitudinal direction of the precursor or CMS fiber. The flat sheet CMS membranes, which were derived from the “dense” precursor membranes, had a higher He/N_2_ selectivity of 10.6, while CMS hollow fibers had a lower selectivity of 7.97, which still suggested that no nanoscopic defects were formed during the formation of the CMS hollow fiber membranes packed module (Figure S24, Supporting Information). Multiple strands of the CMS hollow fibers (3–10 fibers) were packed into the stainless‐steel fitting (Swagelok®) module to increase the membrane surface area per device volume. CMS hollow fibers showed excellent mechanical stability under module preparation showing the scalability of this approach, which is not easily acquired in other inorganic‐based membranes (e.g., zeolites or metal‐organic frameworks).^[^ ^31^ ^]^

For the direct liquid‐phase separation of hexane isomers, the mixture of hexane isomers is fed through the membrane upstream (typically in the shell‐side), while the draw solvent was continuously flowing in the membrane downstream (typically in the bore‐side) as shown in Figure S25 (Supporting Information). For mixture separations, the stage‐cut was maintained below 10^–4^. Choosing the right draw solvent is critical for enabling the forward osmosis type separations. The different draw solvents generate different chemical potential and osmotic pressure gradients in the setup (Note S4 and Figure S26, Supporting Information). The draw solvent should be miscible with the feed mixture and, more importantly, should minimize the back‐diffusion to the membrane upstream. In this context, 1,3,5‐trimethylbenzene (mesitylene, 7.5 Å) and 1,3,5‐triisopropylbenzene (TIPB, 8.5 Å) were chosen as candidates for the draw solvent. We first measured the single component permeability of the hexane isomers in an OSFO setup (**Figure** 3B) by having pure hexane liquid upstream and pure draw solvents downstream to maximize the osmotic pressure gradient. However, the single component OSFO experiments with mesitylene as the draw solvent showed significant back‐diffusion (or back‐permeation) toward membrane upstream, as shown in Figure S27. The back‐permeance of mesitylene was one‐tenth of that of *n*‐hex since the kinetic diameter of the mesitylene was larger but still comparable to the ultramicropore aperture in 6FDA−DAM CMS membranes. We further measured the equilibrium isotherm of mesitylene for 6FDA−DAM CMS (Figure S27), which revealed a noticeable uptake in the CMS membrane. In contrast, TIPB did not show any back permeation to the membrane upstream due to its larger kinetic diameter (8.5 Å) than the pore aperture of the CMS membrane calculated from the graphite model (6.3 Å). TIPB showed an order of magnitude less back‐permeation than mesitylene; therefore, we chose TIPB as the draw solvent for all OSFO experiments. As shown in **Figure** 3B, the single component molar flux generally followed the trends in the size of the hexane isomers, which corresponds to the diffusion selectivities obtained from the vapor sorption study. The ideal selectivity of *n*‐hex/2,3‐DMB was 182, and 2‐MP/2,3‐DMB was 21.5, illustrating the potential of the 6FDA derived CMS for membrane‐based separations for all classes of hexane isomers. Notably, the small error bars in the single‐component permeation results suggest consistent and reproducible solvent permeation results using 6FDA‐polyimide based CMS membranes.

Permeation of organic liquids in the mixture could differ from that of pure components due to increased molecular interactions between molecules, namely, cross‐effects such as the frictional coupling effect.^[^ ^60^, ^61^, ^62^ ^]^ In this context, two binary mixtures containing 2,3‐DMB and other isomers were used to demonstrate the mixture permeation behavior (**Figure** 3C,D). For the *n*‐hex/2,3‐DMB equimolar binary pair, the average molar flux of *n*‐hex was 2.65×10^–3^ mol m^–2^ hr^–1^, whereas that of 2,3‐DMB was only 1.41×10^–4 ^mol m^–2^ hr^–1^ (average permselectivity of 40.2), indicating the effective separation of hexane isomers in the mixture feed with the OSFO setup. Similarly, for the 2‐MP/2,3‐DMB equimolar binary pair, 2‐MP and 2,3‐DMB had an average molar flux of 3.01×10^–4^ mol m^–2^ hr^–1^ and 2.18×10^–5^ mol m^–2^ hr^–1^, respectively (average permselectivity of 13.8). Therefore, the CMS membrane fabricated in this work can fractionate all isomers from each other in mixture separations. As probed by our diffusion analysis, the permselectivity for 2‐MP/2,3‐DMB mostly originated from the entropically limited selection process. In particular, for all cases, the initial detection of 2,3‐DMB after the permeation of light species (e.g., *n*‐hex or 2‐MP) was delayed for 3 to 4 days, which corresponds to a significantly lower diffusivity of 2,3‐DMB that affects the longer time to reach the steady‐state membrane operation. It is also interesting to note that both 2‐MP and 2,3‐DMB permeated ten times faster in a binary mixture than in a single component, while *n*‐hex permeated at a similar rate, suggesting strong coupling effects in binary mixture feeds.

**Figure 4:**
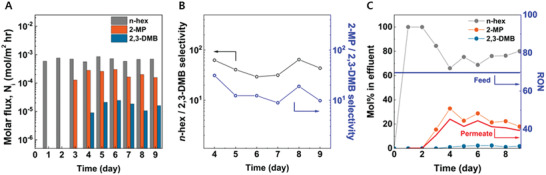
Results of OSFO experiment at room temperature with 6FDA−DAM CMS hollow fiber membranes pyrolyzed at 500 °C (draw solvent: pure TIPB). A) Ternary mixture permeation data versus experimental time. B) Selectivity of 2,3‐DMB over *n*‐hex or 2‐MP. C) Mol fraction change in feed and permeate during the experiments, together with calculated RON.

We further extended our findings to investigate more realistic feed mixtures with equimolar *n*‐hex, 2‐MP, and 2,3‐DMB to ensure the potential of the membrane process for the fractionation of complex mixtures. **Figure** **4** shows the OSFO performance with ternary mixtures plotted against the experimental time. The initial detection of the molecular flux was delayed in the order of *n*‐hex, 2‐MP, and 2,3‐DMB, which was similar to that of binary permeation, with the average molar flux of 6.85×10^–4^, 2.13×10^–4^, and 1.66×10^–5^ mol m^–2^ hr^–1^, respectively, again corresponding to diffusion selectivities. It should be highlighted that ternary permeances were lower than those of the binary OSFO experiments. For instance, *n*‐hex flux has decreased 4‐fold in ternary cases compared to binary cases, indicating the significant coupling effect under complex feed streams. The osmotic pressure gradients of *n*‐hex and 2‐MP across the membrane were similar in binary and ternary OSFO experiments (∼1000 and ∼1,300 bar, respectively). In contrast, the osmotic pressure gradient of 2,3‐DMB in the ternary case was up to 2,100 bar, which was higher than the 1,800 bar calculated from the binary case. These results revealed that a confined and rigid ultramicroporous structure of a 6FDA−DAM CMS hollow fiber membrane created severe competitive permeation of closely‐sized isomers.

It is also notable that the permselectivities (**Figure** 4B) of linear isomer and mono‐branched isomer over di‐branched isomer (average permselectivity of 45.4 and 15.6, respectively) were still maintained, although there should have been a coupling effect among hexane isomers due to friction from molecular interaction between different molecules. That might have been due to the activation energy of diffusion and different confined states of the isomers. As already mentioned, our diffusion analysis revealed that *n*‐hex was relatively easy to diffuse through the micropores of CMS (based on both diffusivity and activation energy of diffusion) compared to 2‐MP and 2,3‐DMB. Textural properties predicted from the molecular simulation results suggest the pore size of 6FDA−DAM is similar to the size of the 2,3‐DMB molecule. The interaction between the pore wall and 2,3‐DMB is dominant compared to the interaction between the di‐branched isomer and *n*‐hex, which indicates a weak frictional coupling and high permselectivity of linear over di‐branched isomer as it diffuses through the pores of the 6FDA−DAM CMS membrane. The rigidity of the confined pore structure creates additional swelling resistance and competition of permeates. Although the permeance of each isomer decreased in the ternary mixture feed compared to a single component or binary feed, the coupling effect or higher osmotic pressure gradient did not significantly affect the permeation of 2,3‐DMB, which resulted in high selectivities of linear and mono‐branched isomers over di‐branched isomer.

**Figure** 4C shows the RON calculated based on the permeate mole fraction, showing the decreased RON in the permeate species, which indicates the successful fractionation of hexane isomers via the OSFO process. It should be highlighted that 6FDA−DAM CMS hollow fiber shows separation performance surpassing zeolite membranes, even without external driving force (Table S7). For instance, beta zeolite tubular membrane on *α*‐alumina support shows *n*‐hex flux similar to CMS fiber membrane reported in this study when vapor permeation (100 to 170 °C) with an equimolar quaternary mixture (*n*‐hexane, 3‐methylpentane, 2,2‐dimethylbutane, 2,3‐dimethylbutane) are used.^[^ ^63^ ^]^ However, the selectivity of linear over di‐branched isomer is much higher in the OSFO process. Previously reported H‐ZSM‐5 tubular membrane supported by stainless steel substrate^[^ ^64^ ^]^ shows higher permeance of *n*‐hex in both vapor permeation and pervaporation, however, only demonstrating the selectivity between the linear and di‐branched hexanes. Both of the zeolite membrane cases take advantage of the phase change of hexane isomers, therefore the OSFO process working on direct liquid phase contact with membranes could pose opportunities for reduction in process energy. Considering the relatively thick membrane layer in the current CMS hollow fiber (50–80 µm), further improvement in molar fluxes are expected for sub‐micron thick CMS membranes fabricated with either thin‐film composite structure^[^ ^65^ ^]^ or post‐treatment of the polymer precursors.^[^ ^66^ ^]^ To demonstrate the possibility of reducing the membrane thickness, we fabricated a thin‐film composite hollow fiber membrane using macroporous *α*‐alumina support by dip‐coating of 6FDA‐DAM and pyrolysis to produce a thin layer of CMS. As illustrated in Figure S28, the membrane thickness of the composite fiber (i.e., CMS layer on *α*‐alumina support) was found to be less than 2 microns. With this approach, a defect‐free composite hollow fiber membrane was formed (He/N_2_ selectivity of 6.67). We demonstrate the up to 100x increase in liquid permeance for both *n*‐hex and 2,3‐DMB using 6FDA‐DAM CMS on alumina fiber (Figure S28).

## 3 Conclusion

In this work, we reported the ultramicroporous carbon hollow fiber membranes synthesized with 6FDA‐polyimides could directly separate liquid‐phase hexane isomers based on the size and shape of the molecules via organic solvent forward osmosis (OSFO) with sub‐0.1 nm size resolution. Organic solvent nanofiltration (OSN) has been a popularized concept for liquid hydrocarbon separation, but the membranes used in OSN allowed the only separation between “solute”‐“solvent” molecules due to the lack of molecular specificity.^[^ ^6^ ^]^ Previous studies on organic solvent forward osmosis (OSFO) were demonstrated in the molecular cut‐off regime used in the OSN processes.^[^ ^33^, ^34^ ^]^ In fact, previous literature on the OSFO process uses the “draw solute” to induce osmotic pressure gradient in the membrane downstream.^[^ ^33^, ^34^, ^67^, ^68^, ^69^ ^]^ However, the use of “draw solute” suffers from the reverse solute flux that might contaminate the mixture feeds, and this approach leaves concern of membrane fouling. Organic solvent reverse osmosis (OSRO) has been widely demonstrated with different membrane materials, including polymers and carbons, showing effective direct liquid separation with excellent molecular selectivities.^[^ ^8^, ^9^, ^10^, ^11^, ^12^, ^13^, ^14^, ^15^, ^16^ ^]^ However, OSRO suffers from the high transmembrane pressure that sets concerns on membrane materials selection and other utilities.^[^ ^35^ ^]^ In contrast, OSFO demonstrated in this work shows the molecular specificity capable of “solvent”‐“solvent” separations, which was only possible in the OSRO process with sub‐0.1 nm resolution. It should also be noted that the utilization of a large molecular weight “draw solvent” generates sufficient osmotic pressure gradient to promote permeation successfully, with no back‐diffusion. The extracted species from feed to downstream require additional separation from the draw solvent; however, the recovery of light species from the heavy draw would require less energy, as shown in prior studies in conventional forward osmosis processes^[^ ^70^ ^]^ and our process calculations (Table S8, Supporting Information).

It is worth reminding that the CMS membranes show the ultramicropore dimensions sized similarly with the kinetic diameter of the hexane isomers that allowed the diffusional cut‐off of *n*‐hex, 2‐MP, and 2,3‐DMB. The kinetic separation of molecules based on the molecular mobility of the two isomers due to the shape differences, namely expressed as shape selectivity, was demonstrated in mono‐branched/di‐branched pair. Motivated by these results, we performed a scale‐up approach for the direct liquid‐phase separation of hexane isomers based on the hollow fiber membranes of the 6FDA−DAM precursor. OSFO with binary and ternary mixture showed the maximized transmembrane osmotic pressure gradient, which resulted in the separation of hexane isomers at room temperature. We believe that designed membranes having different microporosity and functionalities can be used to tune the molecular selectivities of the organic solvent separation process – for instance, selective sorption of chemical species, which is utilized in zeolitic membranes for the “defect reparation”, could be employed in CMSs to finely tune their ultramicroporous region.^[^ ^71^, ^72^ ^]^ Although we utilize the hexane isomers to illustrate our approach in this study, we believe our stable carbon molecular sieve OSFO membrane platform can be applied across a wide range of difficult organic liquid separations. For example, Thompson et al.^[^ ^35^ ^]^ recently reported rationally designed polymeric membranes with intrinsic microporosity capable of fractionating light crude oil with highly complex hydrocarbon mixtures. Therefore, a more efficient way of synergistically combining the benefits of both materials and process will make large‐scale separation process more energy‐efficient.

## 4 Experimental Section

For all the experiments, see the Supporting Information.

## Conflict of Interest

The authors declare no conflict of interest.

## Supporting information

Supporting Information

## Data Availability

The data that support the findings of this study are available from the corresponding author upon reasonable request.
