# Supplementary material for: Shape‐Selective Ultramicroporous Carbon Membranes for Sub‐0.1 nm Organic Liquid Separation
Source: Adv Sci (Weinh). 2021 Jul 11;8(17):2004999. doi: 10.1002/advs.202004999 (PMC8425864; doi:10.1002/advs.202004999)
Supplement: Supplementary file 1 — Supporting Information [file ADVS-8-2004999-s001.pdf]

WILEY-VCH

© 2021 Wiley-VCH GmbH

ADVANCED SCIENCE
Open Access

# Supporting Information

for *Adv. Sci.*, DOI: 10.1002/advs.202004999

# Shape-Selective Ultramicroporous Carbon Membranes for Sub-0.1nm Organic Liquid Separation

*Hyeokjun Seo, Sunghyun Yoon, Banseok Oh, Yongchul G. Chung, and Dong-Yeun Koh **

# Supporting Information

## Shape-Selective Ultramicroporous Carbon Membranes for Sub-0.1nm Organic Liquid Separation

*Hyeokjun Seo*, *Sunghyun Yoon*, *Banseok Oh*, *Yongchul G. Chung, and Dong-Yeun Koh **

**Contents**

1. Materials and Methods

2. Figure. S1 to S 28

3. Table S1 to S8

4. Supplementary Note. S1 to S4

5. References

**Materials and Methods**

**Materials**

4,4’-(Hexafluoroisopropylidene)diphthalic Anhydride(6FDA, 98.0%, TCI chemicals), 2,4,6-trimethyl-1,3-phenylenediamine (DAM, 98.5%, TCI chemicals), 3,5-diaminobenzoic acid(DABA, 98%, Sigma-Aldrich), 4,4'-Biphthalic Anhydride (BPDA, 98%, TCI chemicals) were purified by vacuum sublimation before use. Acetic anhydride (AcAn, >99%, Sigma-Aldrich), 3-methylpyridine (beta picoline, 99%, Sigma-Aldrich) were further dried by zeolite 3A sieve before use. Alumina (AES-22S, Sumitomo), polysulfone (Ultrason® S6010, BASF), dimethylacetamide (DMAc, 99%, Samchun), polyethyleneglycol (PEG 200, Samchun), BYK-150 (BYK), magnesium hydroxide ($Mg(OH)_2$, 95.0%, Samchun), N-methyl-2-pyrrolidone (NMP, 99%, Alfa Aesar), tetrahydrofuran (THF, 99.9%, Samchun), lithium nitrate ($LiNO_3$, 99%, Alfa Aesar), ethanol (EtOH, 99.9%, Daejung), methanol (MeOH, 99.5%, Daejung) and organic solvents (*n*-hexane, 97%, Alfa Aesar; 2-methylpentane, 99%, Alfa Aesar; 2,3-dimethylbutane, 98%, Alfa Aesar; 1,3,5-trimethylbenzene, 97%, Arcros-organics; 1,3,5-triisopropylbenzene, 95%, TCI chemicals) were used as received.

**Polyimide synthesis**

Three different 6FDA-based polyimides (**Figure S3**) were synthesized by conventional two-step polycondensation reaction. All monomers were purified by vacuum sublimation and solvents were dried with 3A molecular sieve before use. The stoichiometric amounts of dianhydride and diamine monomers were weighed and dissolved in anhydrous NMP. At the former step, polyamic acid with a high molecular weight was produced under $N_2$ purging and stirring at low temperature, after 1 day. Then the polyimide was produced by chemical imidization by means of beta picoline and AcAn as a catalyst and dehydrating agent,

respectively, at ambient temperature. The resultant polyimide (**Figure S3**) was precipitated in MeOH, followed by vacuum drying at 210°C for 1 day.

**Flat sheet membrane casting**

Flat sheet dense membranes of each polyimide were cast on a glass plate with a doctor blade from viscous polymer solution inside a glove bag. A glove bag was saturated with NMP vapor for the slow evaporation of NMP (solvent) from spread solution before casting. After slow evaporation of solvent at 60°C about 1 day, the membrane was removed from the plate and solvent exchanged with deionized (DI) water, MeOH and n-hexane sequentially to remove residual solvent. The membranes were further dried in 120 °C vacuum oven overnight. Physical properties of polyimides are tabulated in **Table S2**.

**Dry-jet/wet-quench hollow fiber spinning**

A schematic diagram about the production of asymmetric hollow fiber membrane is illustrated in **Figure S22**. Among the polyimides in this study, we have selected 6FDA−DAM as a precursor owing to its shape selectivity for hexane isomers, as depicted in the main text. The 6FDA−DAM dope solution was composed of 6FDA−DAM; NMP, THF (solvent); EtOH, $LiNO_3$ (non-solvent). A transparent dope solution was formed by shear mixing for about 1week, followed by manual degassing in a syringe pump (Teledyne Isco, 500D). Then bore fluid was individually loaded into another syringe pump and all the pumps were connected with coannular spinneret (Sepra Tek). The spinning conditions in this study are illustrated in **Table S6**. As-spun fibers were solvent exchanged with DI water, MeOH, and n-hexane sequentially, followed by vacuum drying at 120°C to remove any residual solvent.

**Fabrication of thin film composite fiber membrane**

Substrate alumina hollow fiber was fabricated via the spinning method. The dope solution composed of DMAc (20.7 wt%), $Al_2O_3$ (70 wt%), polysulfone (6.5 wt%), PEG 200 (2.0 wt%), BYK 150 (0.5 wt%), and $Mg(OH)_2$ (0.3 wt%) was stirred 72h at 80 °C to form homogeneous solution, and then spun to coagulation bath with bore liquid (water). The flow rate of dope solution and bore liquid were 20 g $min^{-1}$ and 16 ml $min^{-1}$, respectively, and as-spun fiber was sintered at 1150 °C for 1h. Thin film composite fiber composed of 6FDA–DAM skin layer and $\alpha$-alumina substrate fiber was fabricated via dip-coating. The polymer was dissolved in THF and 8wt% dope solution was utilized for dip-coating. As-casted polymer layer was dried at room temperature for 12 h, then at 120 °C vacuum oven for 12 h before conducting pyrolysis.

**Fabrication of CMS membranes (pyrolysis)**

A 60×7.5cm quartz plate was employed to load flat sheet membranes or fibers into the tube furnace (SH scientific, SH-FU-80LTG-0M). The oxygen concentration in the quartz tube was analyzed via an oxygen analyzer (Cambridge Sensotec Ltd., Rapidox 1100-ZR) and ultra-high purity (UHP, 99.999%) argon was provided to the tube using a mass flow controller (Omega Engineering) to hold the oxygen level around 30ppm before every pyrolysis process. In this study, all the precursor polymer membranes were pyrolyzed at a fixed soaking temperature of 500 °C (ramping protocol; RT-250°C: 10°C $min^{-1}$, 250-480°C: 3°C $min^{-1}$, 480-500°C: 0.2°C $min^{-1}$, 2hr soaking at 500°C, natural cooling to RT). After each pyrolysis, the quartz tube and plate were washed with acetone, followed by bakeout in ambient air at 800°C to remove any pyrolytic residue.

**Membrane module formation and single gas permeation experiment**

As-made flat sheet CMS membranes were mounted into a permeation cell to manipulate single gas permeation experiments. Each membrane was first masked between impermeable aluminum tapes. Then five-minute epoxy (3M, DP-100) was utilized in final masking for complete sealing of the CMS membrane and tape interface. On the other hand, CMS hollow fiber membranes were assembled with Swagelok® stainless-steel fittings and the chemical-resistant epoxy (Marine Weld, J-B Weld®) to prevent degradation of epoxy owing to hexane isomers and draw solvent during OSFO experiments, which occurred in the case of five-minute epoxy. Constant volume permeation measurements were conducted at 35°C to confirm the integrity of flat sheet and hollow fiber CMS membranes. In this study, He/$N_2$ selectivity was chosen as an indicator to distinguish Knudsen and defect-free selectivities (**Figure S24**).

**Organic vapor sorption experiment**

The gravimetric vapor sorption isotherms of *n*-hexane, 2-methylpentane and 2,3-dimethylbutane in three different flat sheet CMS membranes were recorded using a VTI-SA+ (TA Instruments) with relative pressure ranging from 0 to 0.85. Flat sheet CMS membranes were cut into small square pieces (i.e. ~1cm x 1cm) and loaded into a quartz pan. The loaded samples were activated at 120°C for 12 hours under the nitrogen flow in the instrument before every sorption experiments start. Organic vapor sorption isotherms and time-dependent uptake isotherms were recorded at 25, 35, and 45°C for three hexane isomers, with varying relative pressure from 0 to 0.85. Flat sheet CMS membranes activated at 120°C for 12 hours were weighed to get initial mass. All hexane isomers are liquid phase at unit activity and the membranes were soaked in each hexane isomers in 20mL vials. Then they were located in an oven at 25, 35, and 45°C for 1 week to ensure the equilibrium, followed by logging weight change and calculation of quantity adsorbed.

**Organic Solvent Forward Osmosis (OSFO) experiment**

6FDA−DAM CMS hollow fibers pyrolyzed at 500°C were employed in OSFO experiments. A schematic diagram of OSFO experiment is shown in **Figure S25**. The feed solution and draw solvent were supplied to the shell side and bore side, respectively, using a 515 HPLC pump (Waters®). The feed composition was varying: single-component OSFO experiments were utilized with pure hexane isomers as feed, while mixture OSFO experiments proceeded with equimolar binary mixture of hexane isomers or equimolar ternary mixture. In all cases, retentate from the shell side of the module was recycled back to the feed reservoir. On the other hand, permeate from the bore side was collected in isolation in sealed bottles rather than reuse, to maintain osmotic pressure gradient of all feed components same over the whole experimental time. Hydraulic pressure values logged with the pump during all experiments were 0 psi (relative pressure). The weight of each hexane isomer in permeate was calculated via GC analysis, followed by normalization of it with time and membrane area to calculate permeance.

**Elemental analysis (EA)**

Elemental analysis of precursor polyimides and CMS membranes was performed with FlashEA 1112 (Thermo Finnigan, Italia) for C, H, N analysis, and Flash 2000 series (Thermo Scientific) for oxygen analysis. To determine the exact composition of pure materials, polyimides were dried at 120°C overnight before each analysis, and CMS membranes were analyzed right after the pyrolysis.

**Thermogravimetric analysis (TGA)**

TGA experiments were performed using TG209 F1 Libra (Netzsch) under an UHP argon flow. The samples were heated up to 800°C with heating rate of 5°C / min.

**Fourier transform Infrared Spectroscopy (FT-IR)**

Attenuated total reflectance Fourier transform infrared spectra were measured on Nicolet iS50 FT-IR spectrometer (Thermo Scientific) under transmittance mode. The range was set at 400 to 4000 $cm^{-1}$ with a resolution of 2$cm^{-1}$.

**X-ray diffraction (XRD)**

XRD patterns of CMS membranes were obtained using SmartLab diffractometer (Rigaku) with Cu K$\alpha$ radiation ($\lambda$=1.5406Å), at a step size of 0.01°.

**Raman spectroscopy**

Raman spectra of samples were recorded using Aramis dispersive Raman spectrometer (Horiba) with 514nm laser.

**Scanning Electron Microscopy (SEM)**

A FEI Nova 230 scanning electron microscope was used to record the geometry and morphology of precursor and CMS membranes. Precursor polymer samples were soaked in hexane, followed by fragmentation in liquid nitrogen to obtain intact morphology in the cross-sectional area. On the other hand, CMS membranes were directly broken. Then the samples were sputtered with gold.

**Transmission Electron Microscopy (TEM)**

A double Cs-corrected TEM (Titan Cubed $G^2$ 60-300, FEI) was utilized to acquire high-resolution TEM images under an acceleration voltage of 300kV. For sample preparation, CMS film was cut with an ultramicrotome (Ultracut EM UC7, Leica, Austria) and mounted on a

TEM grid. ImageJ aided to improve visualization of pore structure in CMS [1].

**Atomic Force Microscopy (AFM)**

The surface images and height profiles of three different CMS membranes were recorded with atomic force microscope (XE-100, Park systems, Korea). The flat sheet CMS samples were prepared exactly the same with the samples utilized in diffusion study (i.e. ~ 1cm × 1cm square plates).

**Physisorption experiments**

77K $N_2$ and 273K $CO_2$ physisorption experiments were performed using ASAP 2020 (Micromeritics). BET (Brunauer-Emmett-Teller) surface areas and 2D-NLDFT (non-local density functional theory) pore size distributions with carbon slit-pore geometry were calculated from 77K $N_2$ isotherm. 87K argon physisorption experiments were performed using 3Flex 3500 (Micromeritics).

**Calculation and comparison of heat duties**

Aspen Plus V8.8 was used for the comparison of theoretical heat duties between three distillation processes: (i) *n*-hex / 2,3-DMB, (ii) *n*-hex / TIPB, (iii) 2,3-DMB / TIPB. The NRTL model was selected for the calculation of physical properties of solvents. The feed compositions were determined based on experimental permeation data with thin film composite fiber membranes. Other modeling parameters were decided to satisfy the product purity as 99.9%, and the results are summarized in **Table S8**.

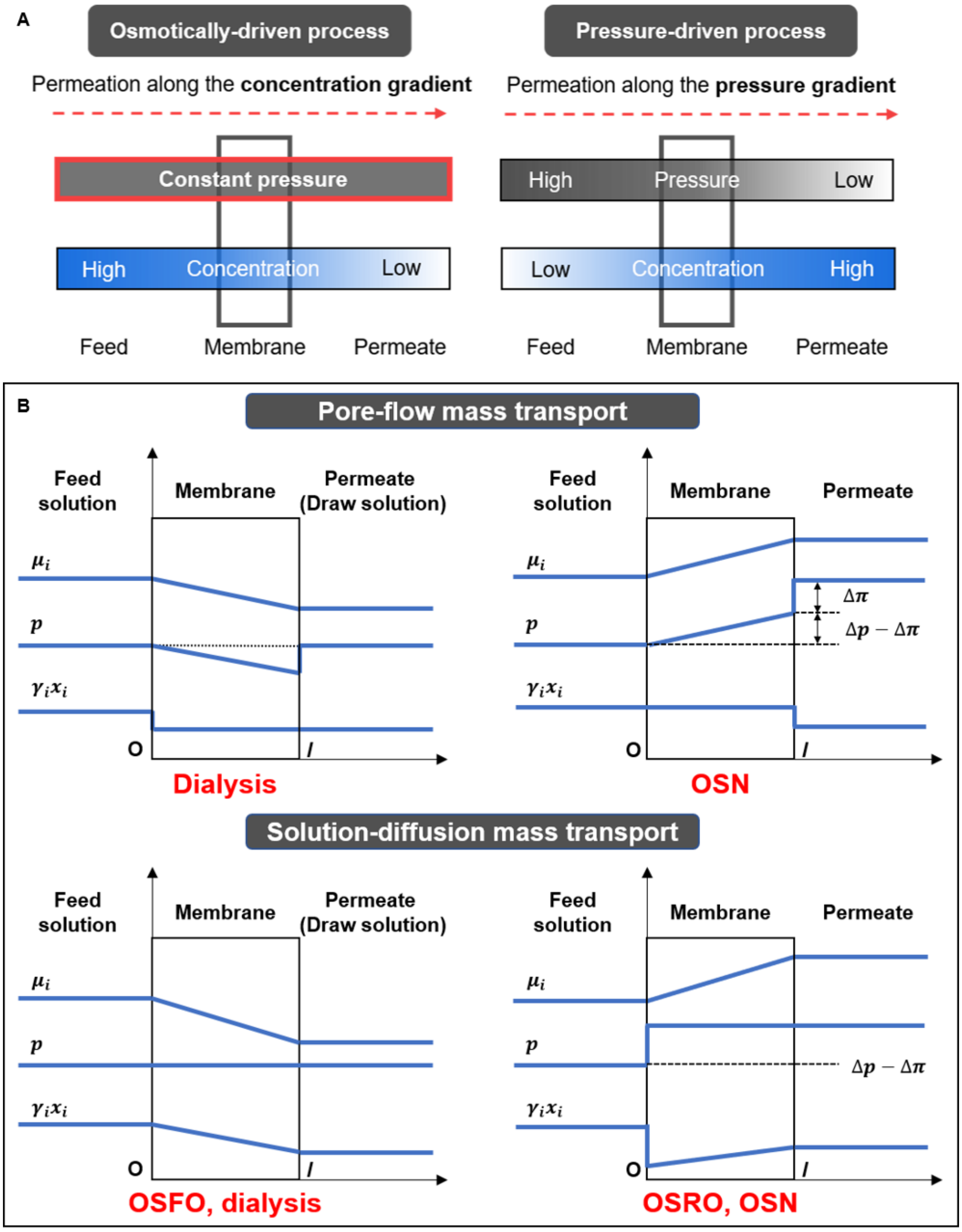


**Figure S1. (A)** Pressure and concentration gradients across the membrane during pressure-driven and osmotically-driven processes, **(B)** Difference of mass transport mechanism between solvent separation modalities : pore-flow transport and solution-diffusion transport

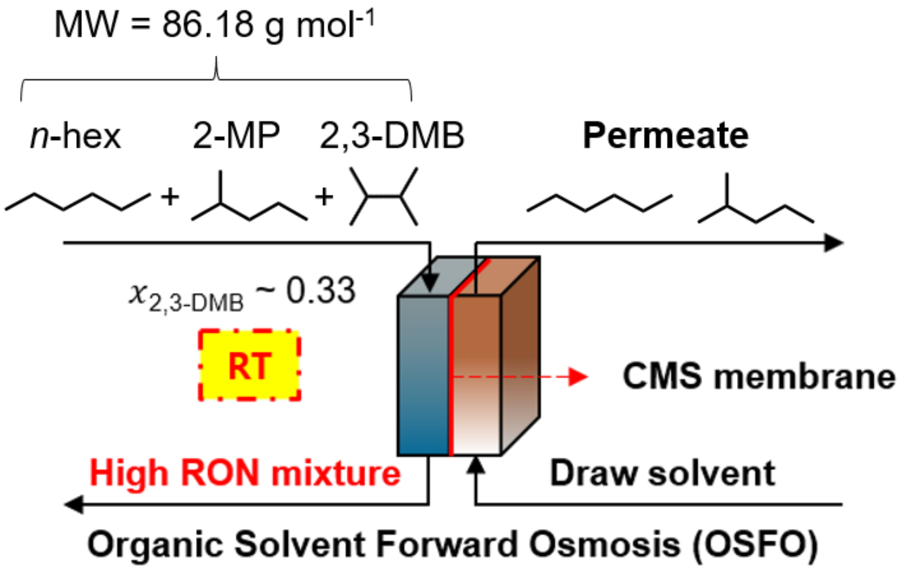


**Figure S2**. Schematic diagram of OSFO process for the separation of liquid mixture of hexane isomers with CMS membrane, utilizing draw solvent for the generation of driving force

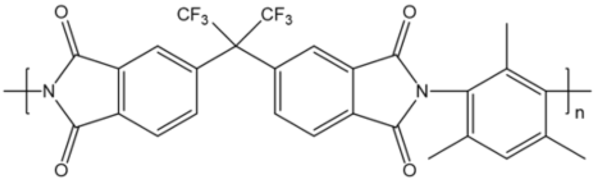

**6FDA−DAM**

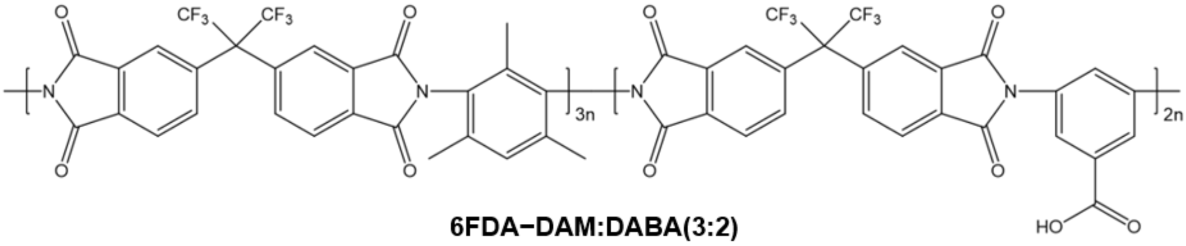

**6FDA−DAM:DABA(3:2)**

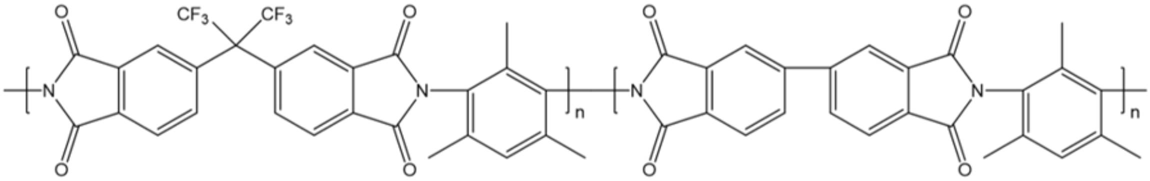

**6FDA:BPDA(1:1)−DAM**

**Figure S3.** Chemical structure, molecular weight and polydispersity index (PDI) of polyimides utilized in this study

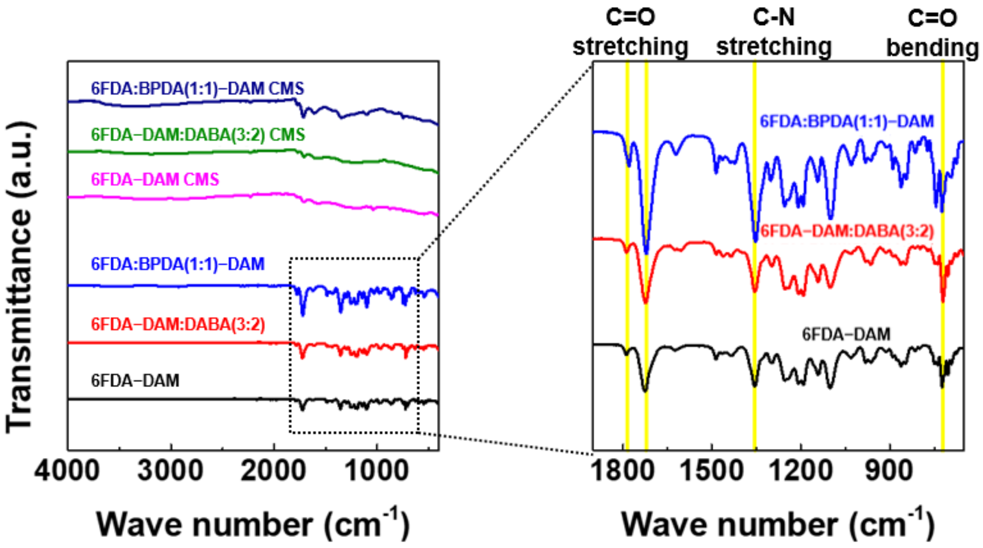


**Figure S4.** FT-IR spectra of precursor polyimides and CMS membranes pyrolyzed at 500°C

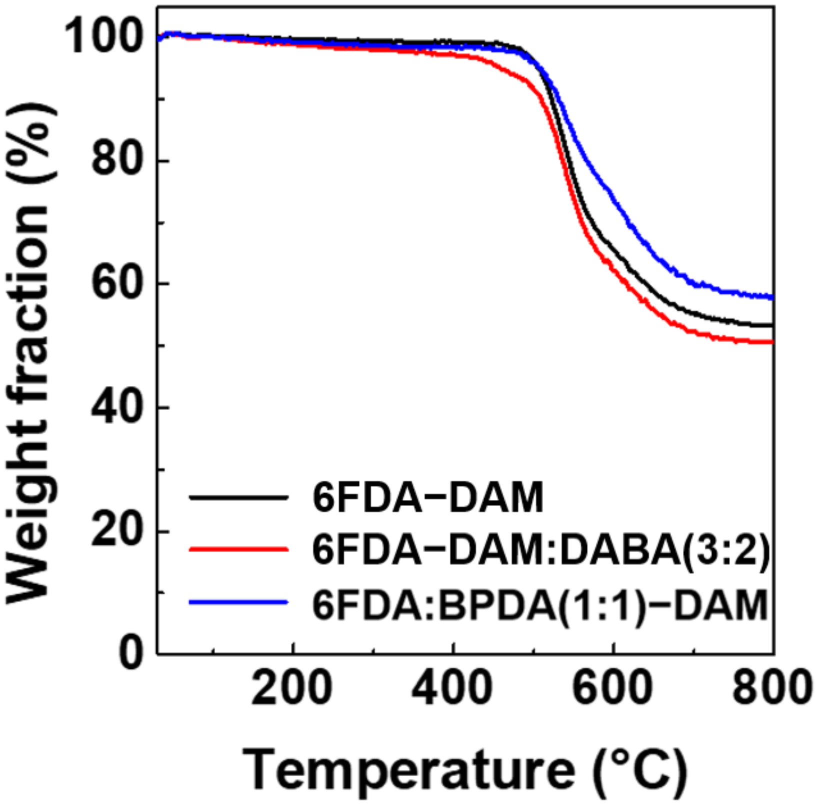


**Figure S5.** TGA profile of precursor polyimides 6FDA−DAM, 6FDA−DAM:DABA(3:2) and 6FDA:BPDA(1:1)−DAM under UHP argon flow

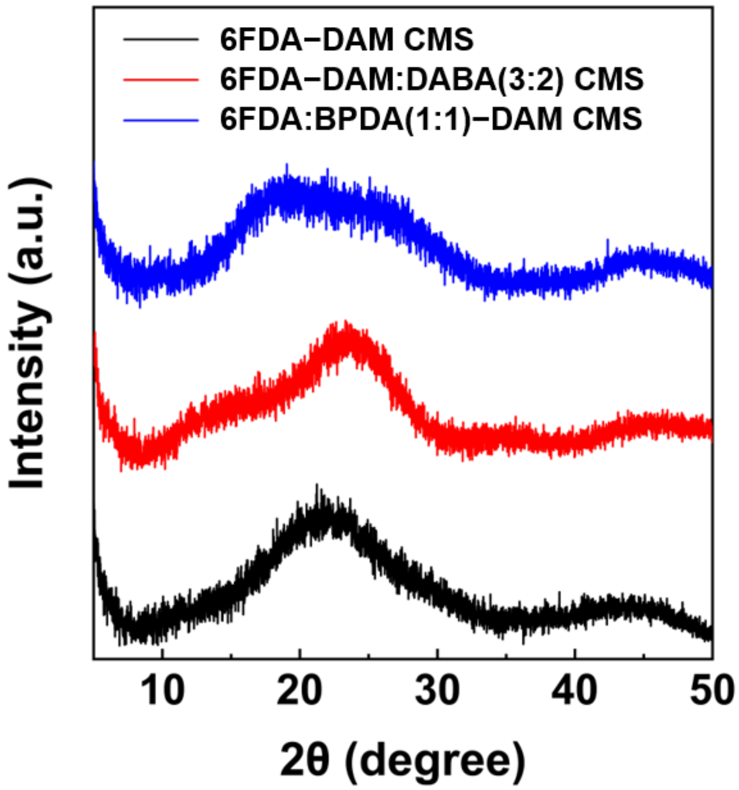


**Figure S6.** X-ray diffraction patterns of CMS membranes pyrolyzed at 500°C

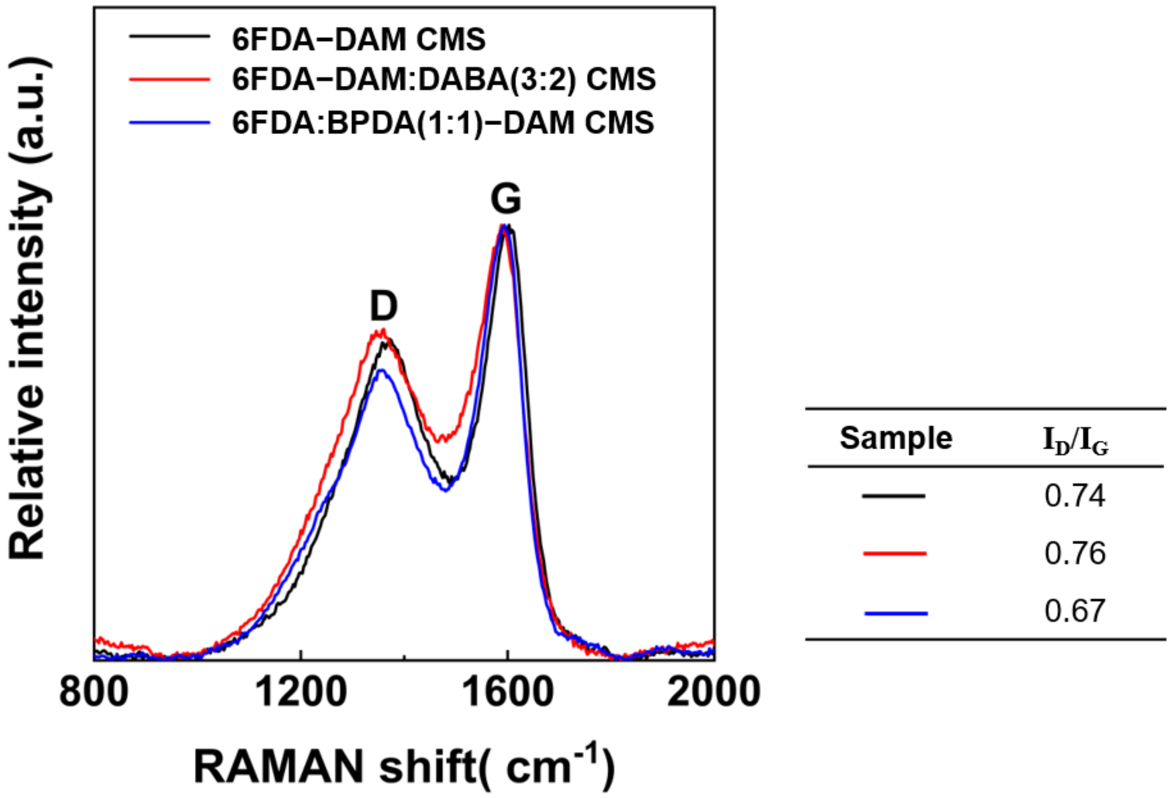


**Figure S7. R**aman spectra of CMS membranes derived from three different polyimides

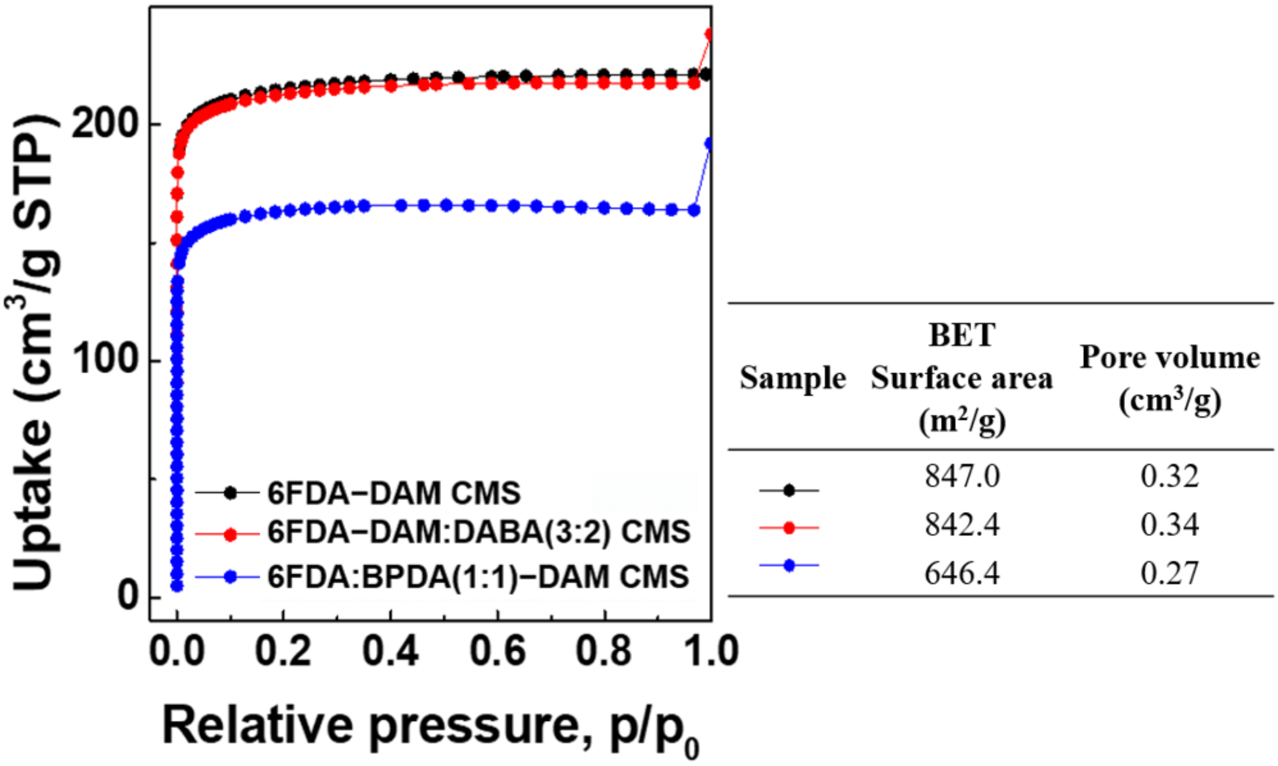


| Sample | BET Surface area ($m^2/g$) | Pore volume ($cm^3/g$) |
|---|---|---|
| —●— | 847.0 | 0.32 |
| —●— | 842.4 | 0.34 |
| —●— | 646.4 | 0.27 |

**Figure S8.** $N_2$ physisorption isotherm at 77K and corresponding BET surface area / pore volume of three different CMS membranes

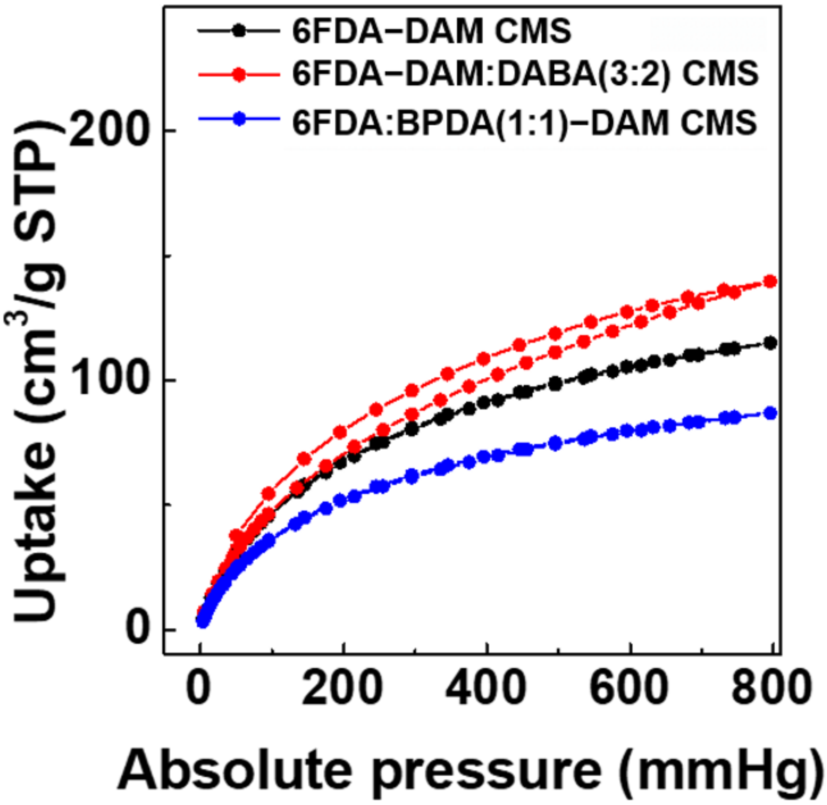


**Figure S9.** $CO_2$ physisorption isotherm at 273K of CMS membranes pyrolyzed at 500°C

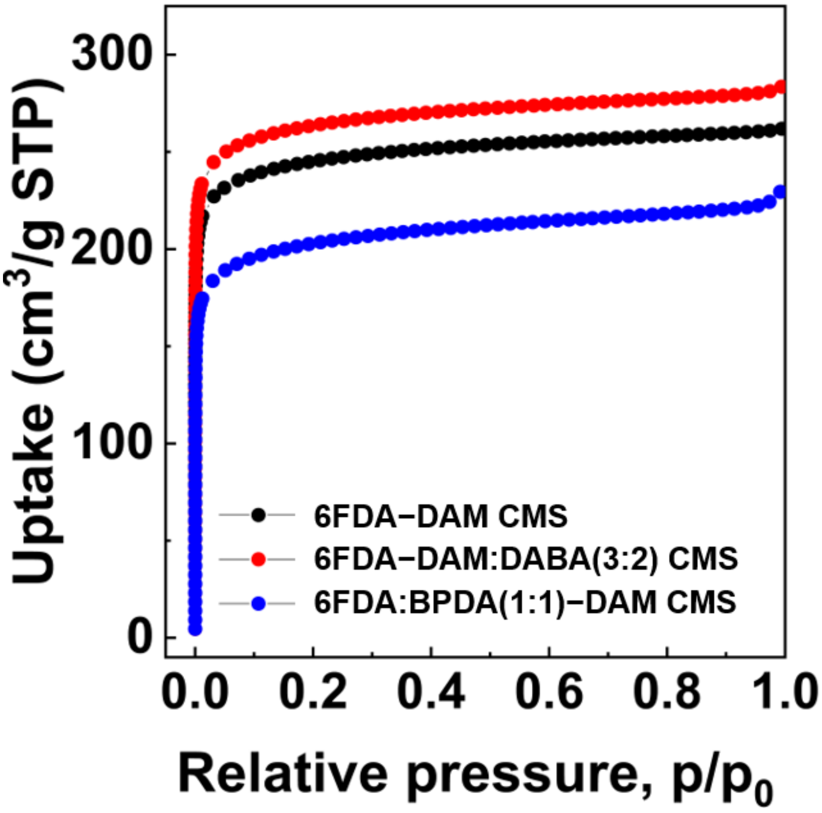


**Figure S10.** Ar physisorption isotherm at 87K of CMS membranes pyrolyzed at 500°C

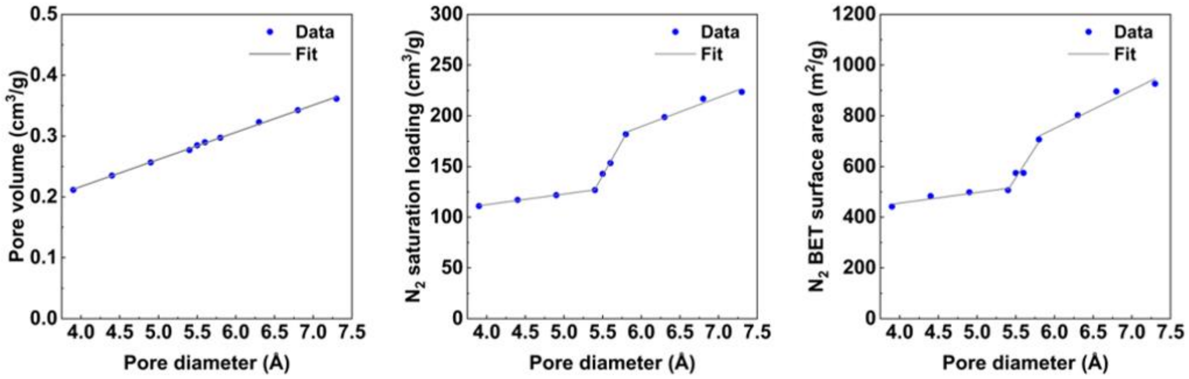


**Figure S11.** Simulated structural features and the fitted linear functions to simulated data as function of simulated pore diameter: **(A)** pore volume ($R_2$ = 0.998), **(B)** $N_2$ saturation loading ($R_2$ = 0.998) and **(C)** $N_2$ BET surface area ($R_2$ = 0.991).

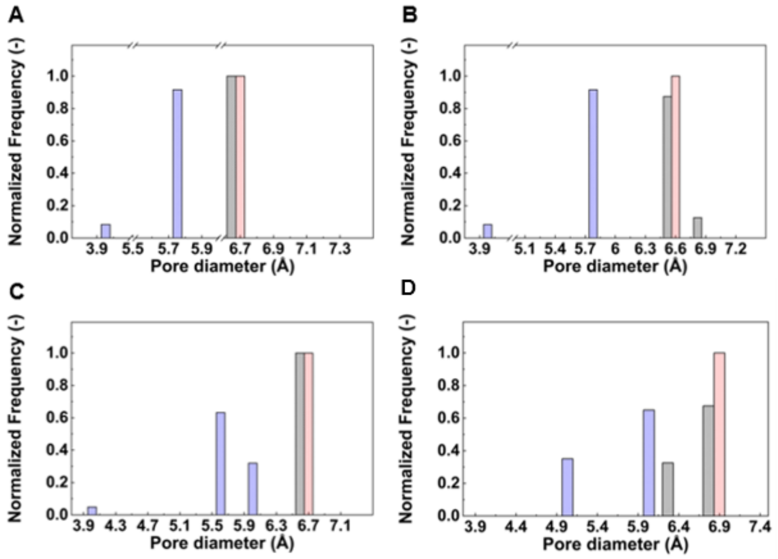


**Figure S12.** Estimated pore size distribution of three different CMS membranes based on $N_2$ physisorption: **(A)** pore diameter interval = 0.2 Å, **(B)** pore diameter interval = 0.3 Å, **(C)** pore diameter interval = 0.4 Å, **(D)** pore diameter interval = 0.5 Å (black; 6FDA−DAM CMS, red; 6FDA−DAM:DABA CMS, blue; 6FDA:BPDA−DAM CMS)

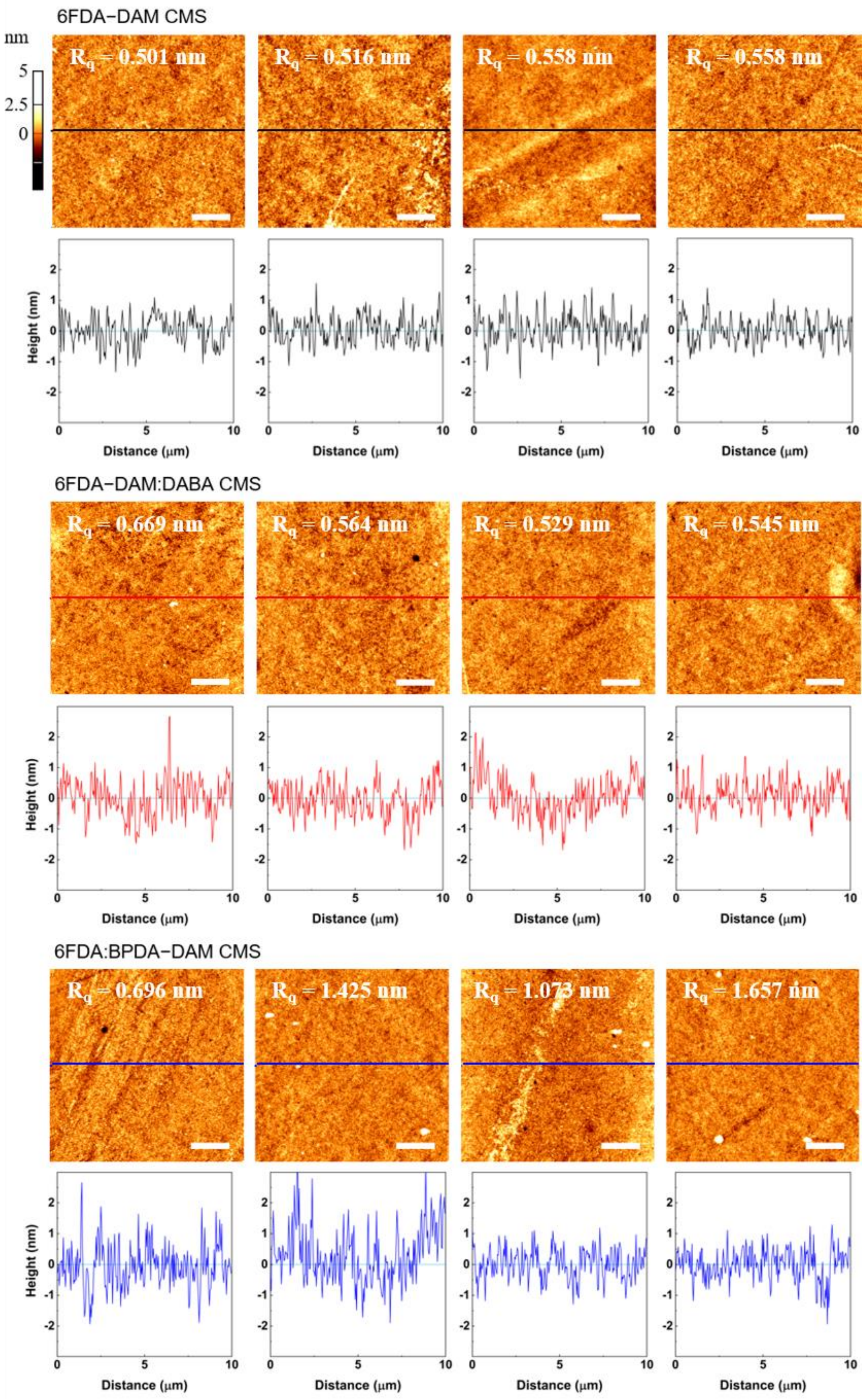


**Figure S13.** AFM images of three different flat sheet CMS membranes collected randomly along the long edge

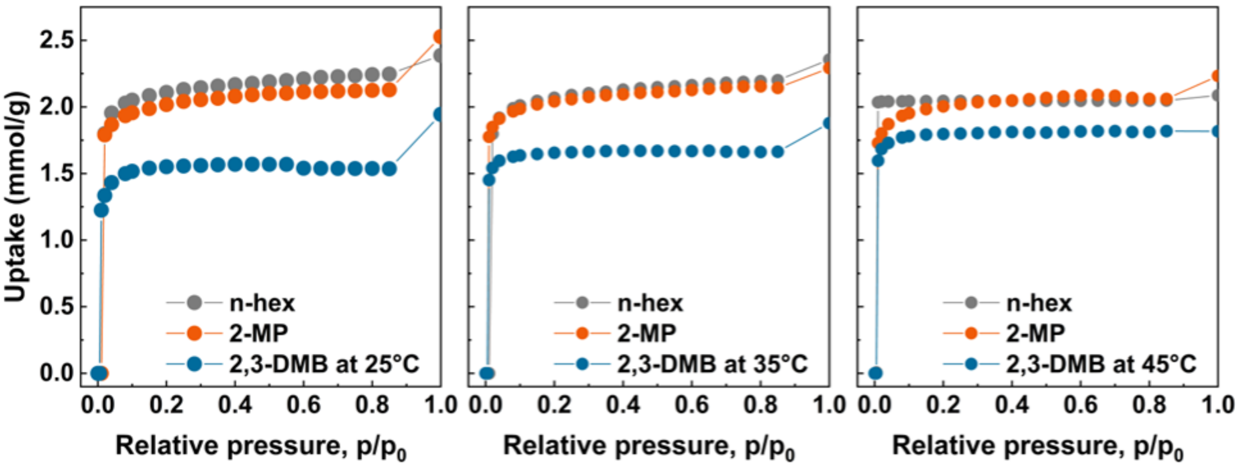


**Figure S14.** Sorption isotherms of hexane isomers at 25, 35 and 45 °C in 6FDA−DAM CMS

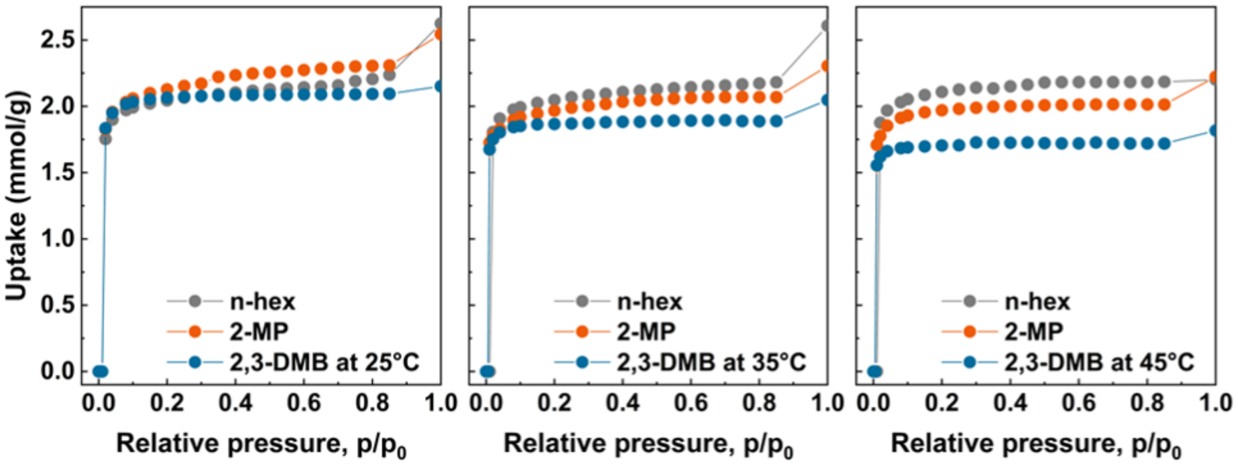


**Figure S15.** Sorption isotherms of hexane isomers at 25, 35 and 45 °C in 6FDA−DAM:DABA(3:2) CMS

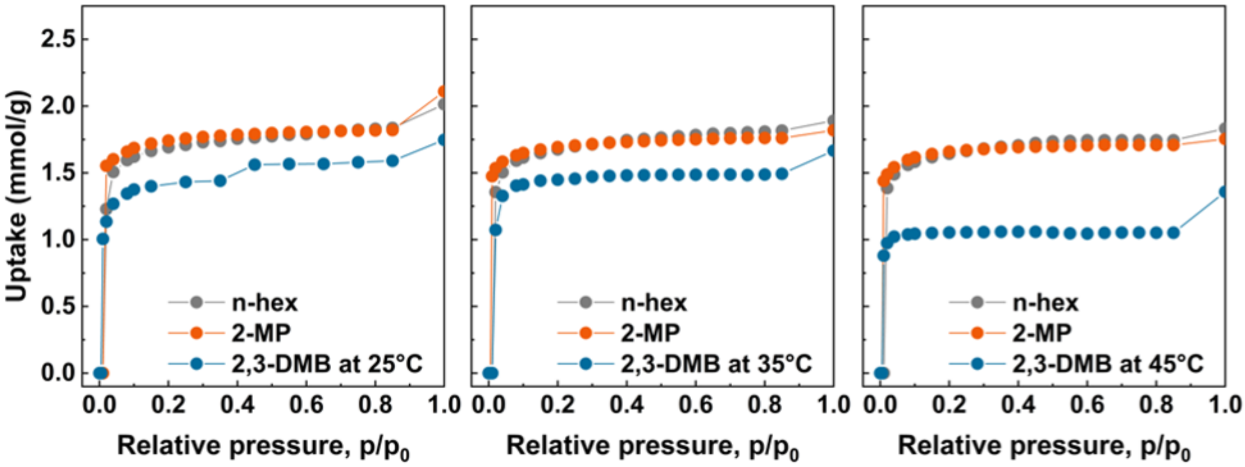


**Figure S16.** Sorption isotherms of hexane isomers at 25, 35 and 45 °C in 6FDA:BPDA(1:1) −DAM CMS

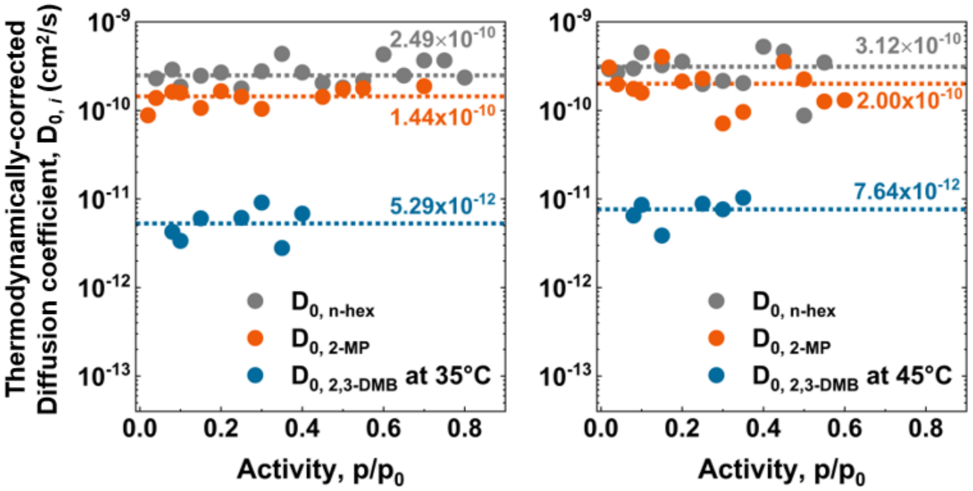


**Figure S17.** Diffusion coefficients of hexane isomers against activity in 6FDA−DAM CMS membrane at 35 °C and 45°C

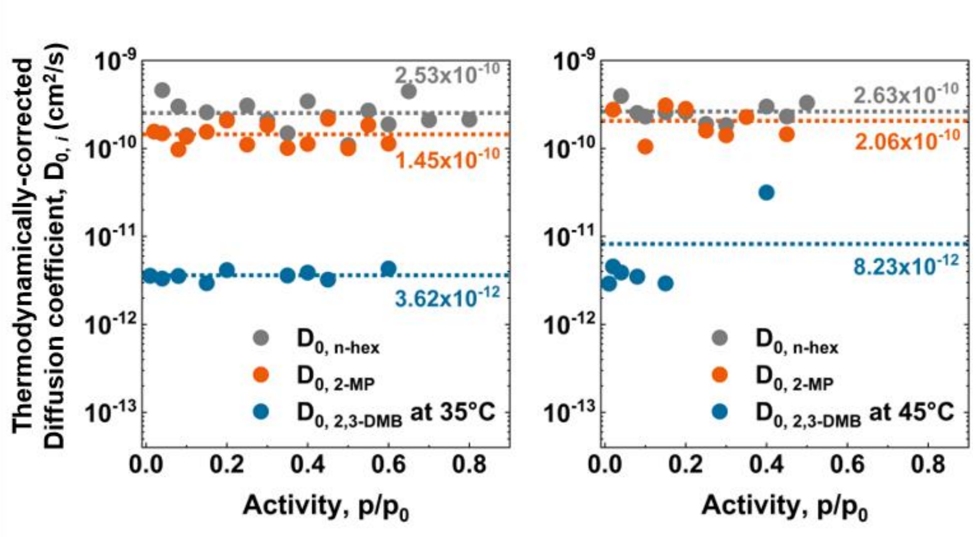


**Figure S18.** Diffusion coefficients of hexane isomers against activity in 6FDA−DAM:DABA(3:2) CMS membrane at 35 °C and 45°C

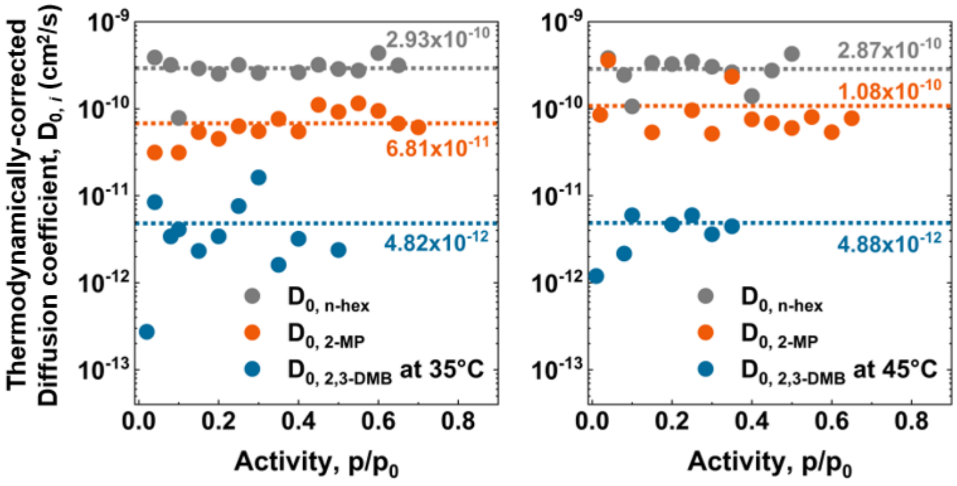


**Figure S19.** Diffusion coefficients of hexane isomers against activity in 6FDA:BPDA(1:1) −DAM CMS membrane at 35 °C and 45°C

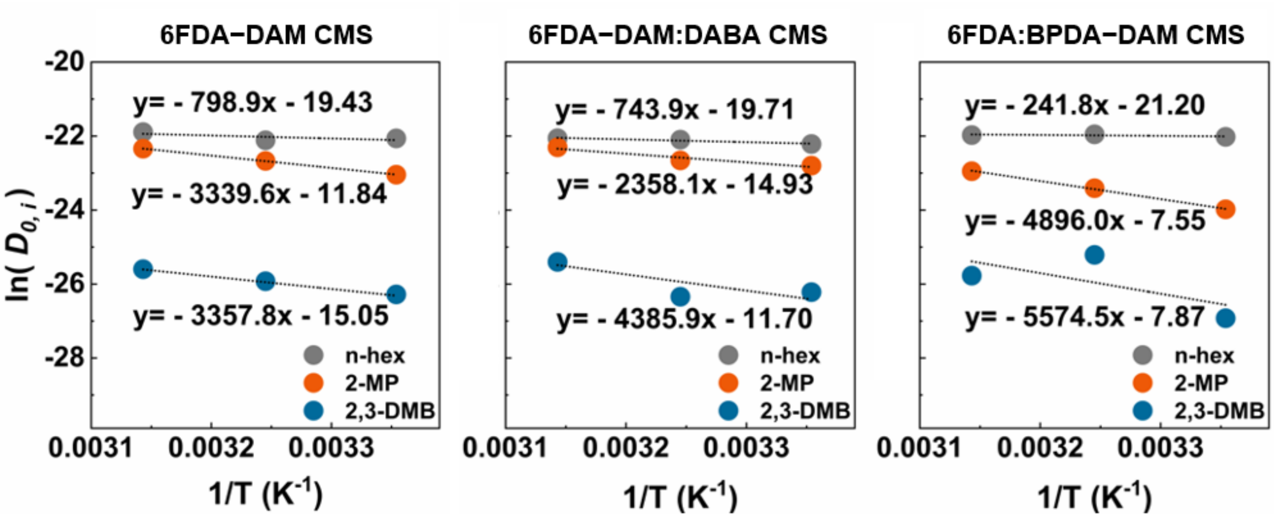


**Figure S20.** Estimation of activation energy of diffusion based on average diffusion coefficients of each isomer at 25, 35 and 45°C via Arrhenius relationship

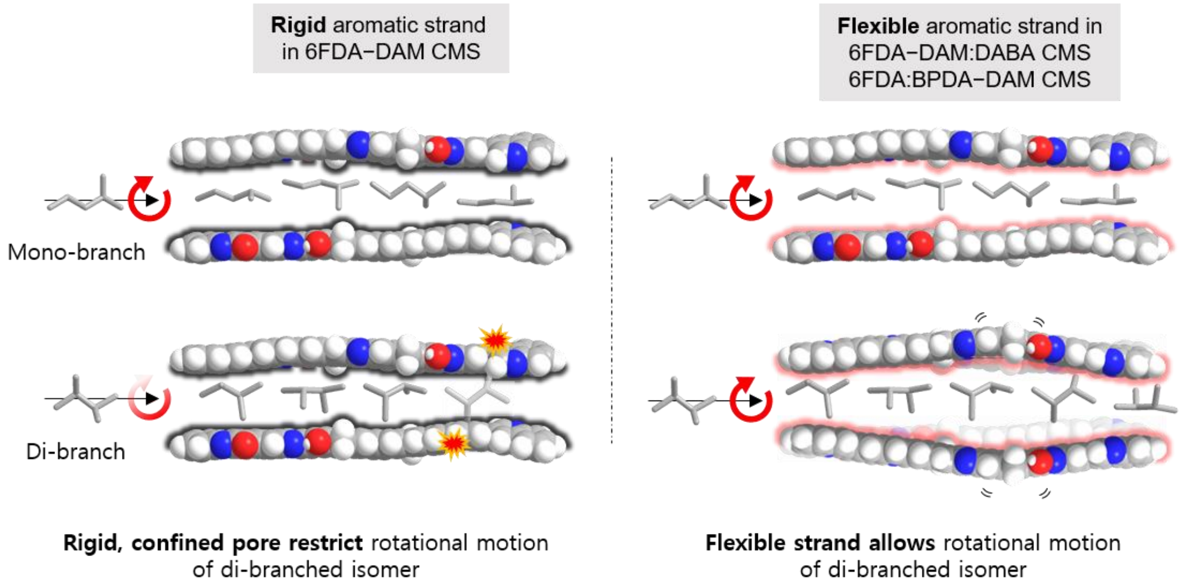


**Figure S21.** Comparison between pore structure composed of aromatic strands with different rigidity, indicating shape-selective transport

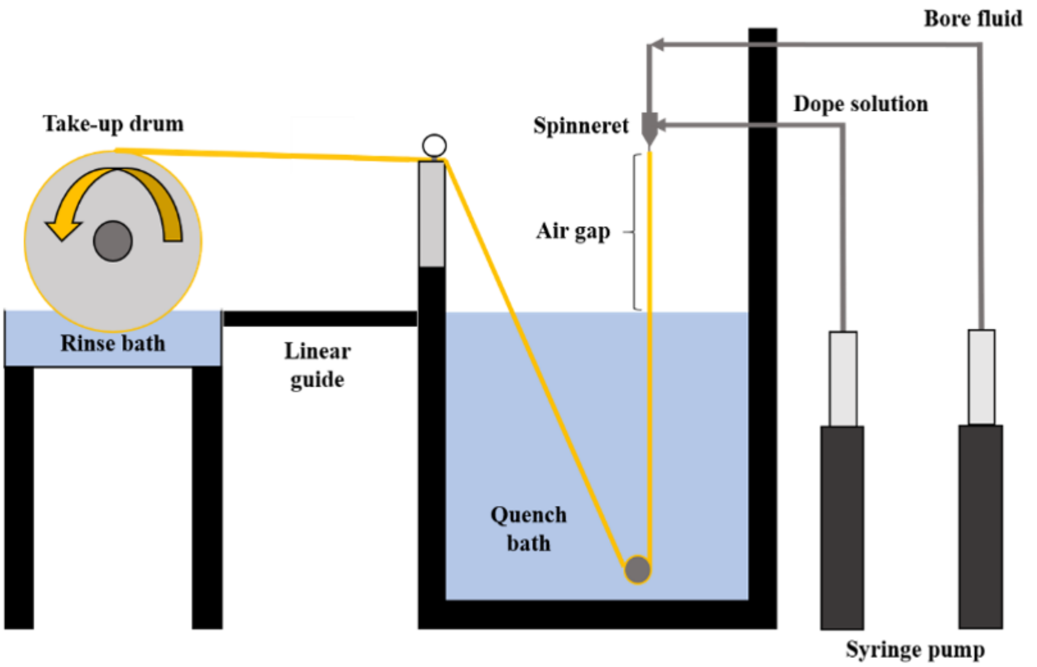


**Figure S22.** Illustration of ‘dry-jet/wet-quench’ hollow fiber spinning process

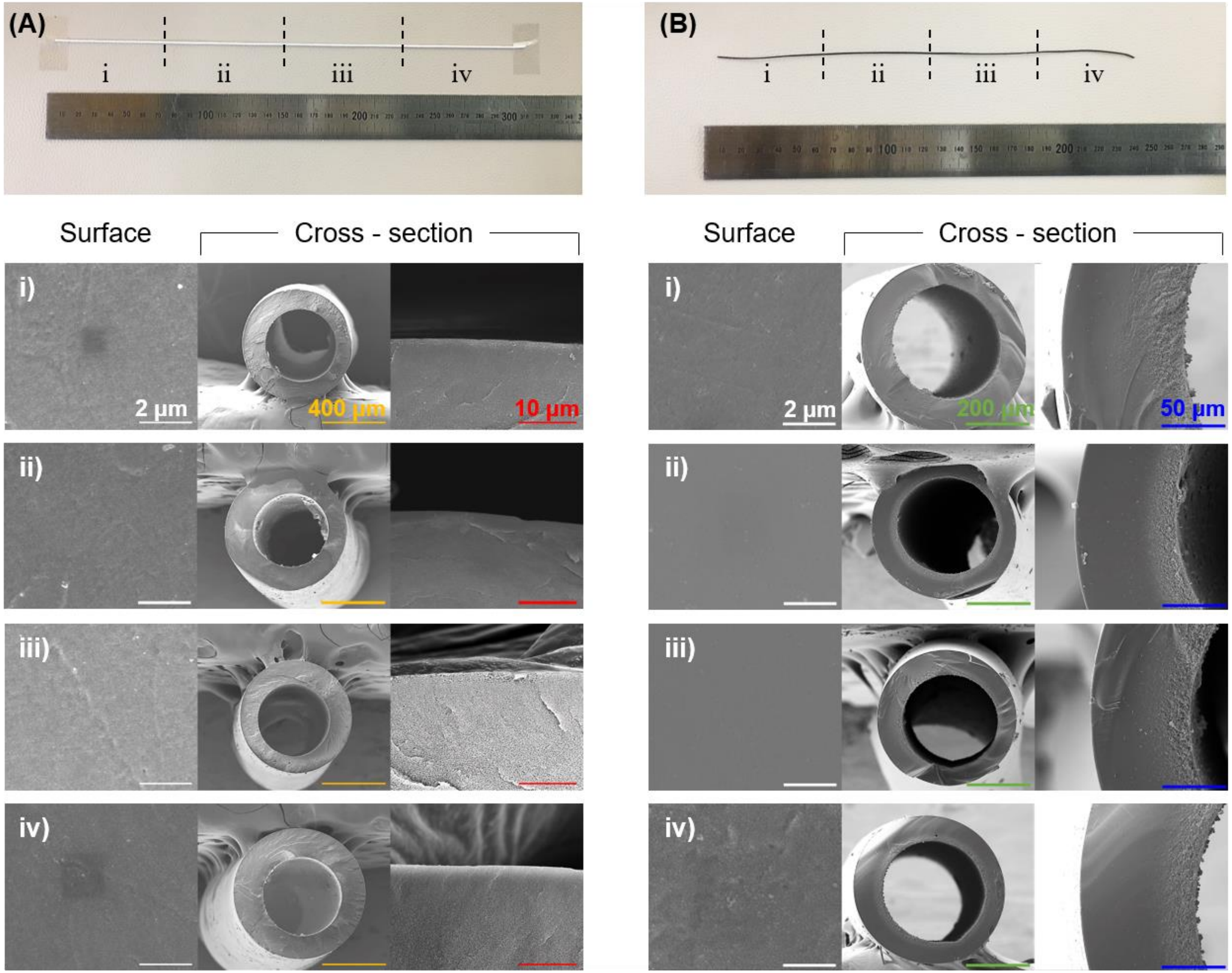


**Figure S23.** Digital image and SEM images of samples taken from i to iv for **(A)** 6FDA–DAM fiber and **(B)** 6FDA–DAM CMS fiber

2 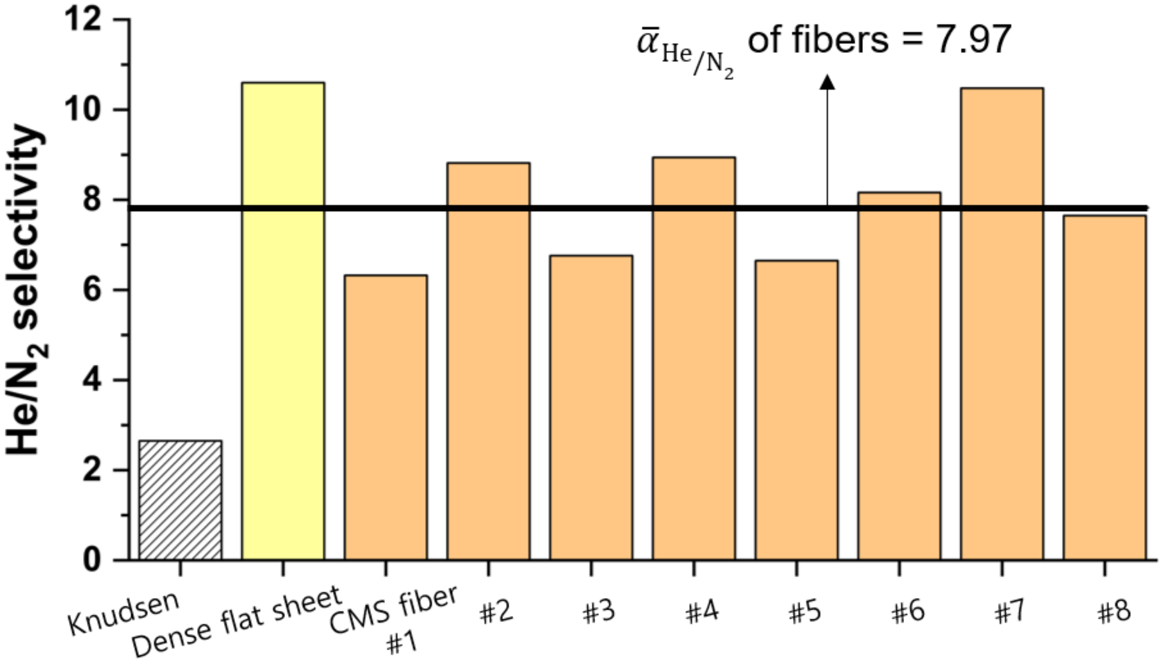


**Figure S24.** He/$N_2$ selectivities in Knudsen flow, dense flat sheet CMS membrane and hollow fiber membranes

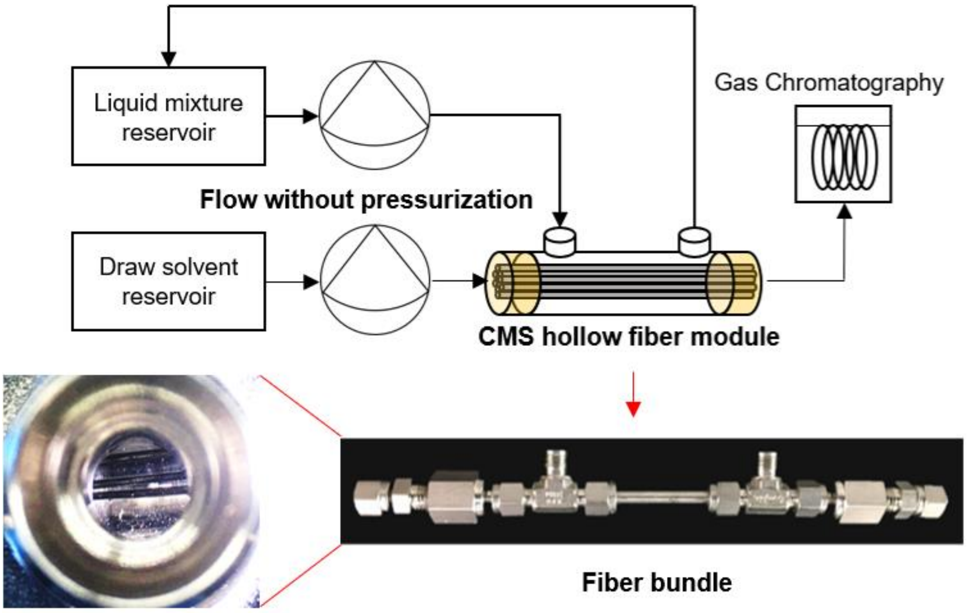


**Figure S25.** Schematic diagram of CMS hollow fiber module and OSFO process

5

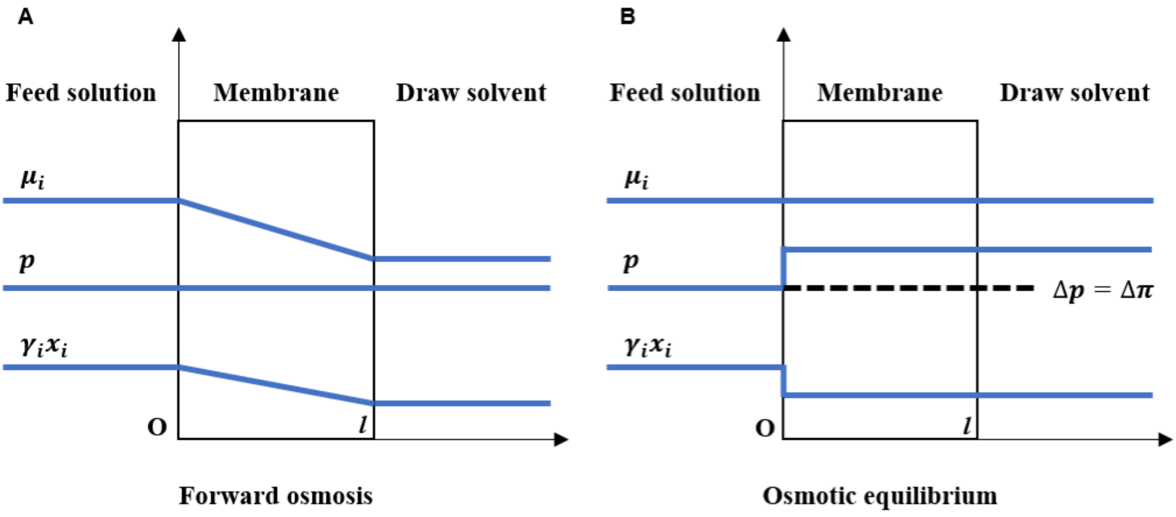


**Figure S26.** Illustration of osmotic pressure gradient as permeation driving force in OSFO process

6

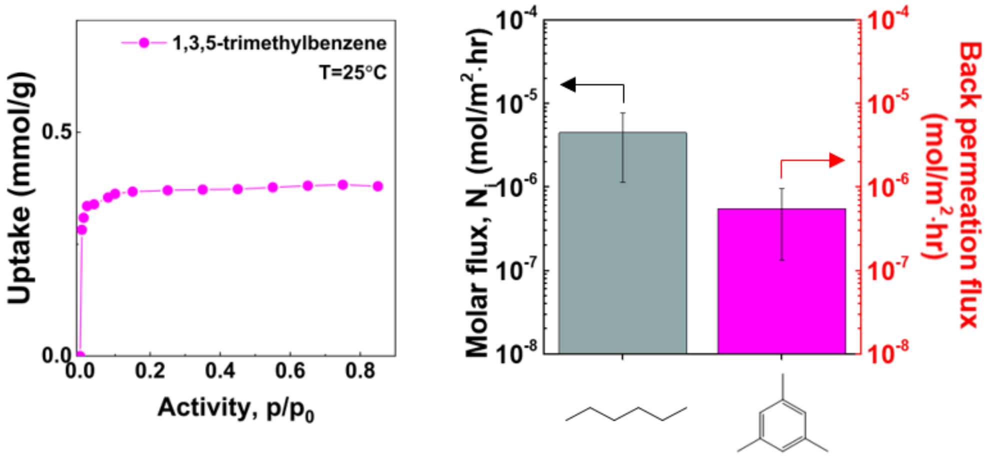


**Figure S27.** Uptake isotherm of 1,3,5-trimethylbenzene in 6FDA−DAM CMS at 25°C and single component OSFO results with 1,3,5-trimethylbenzene as draw solvent

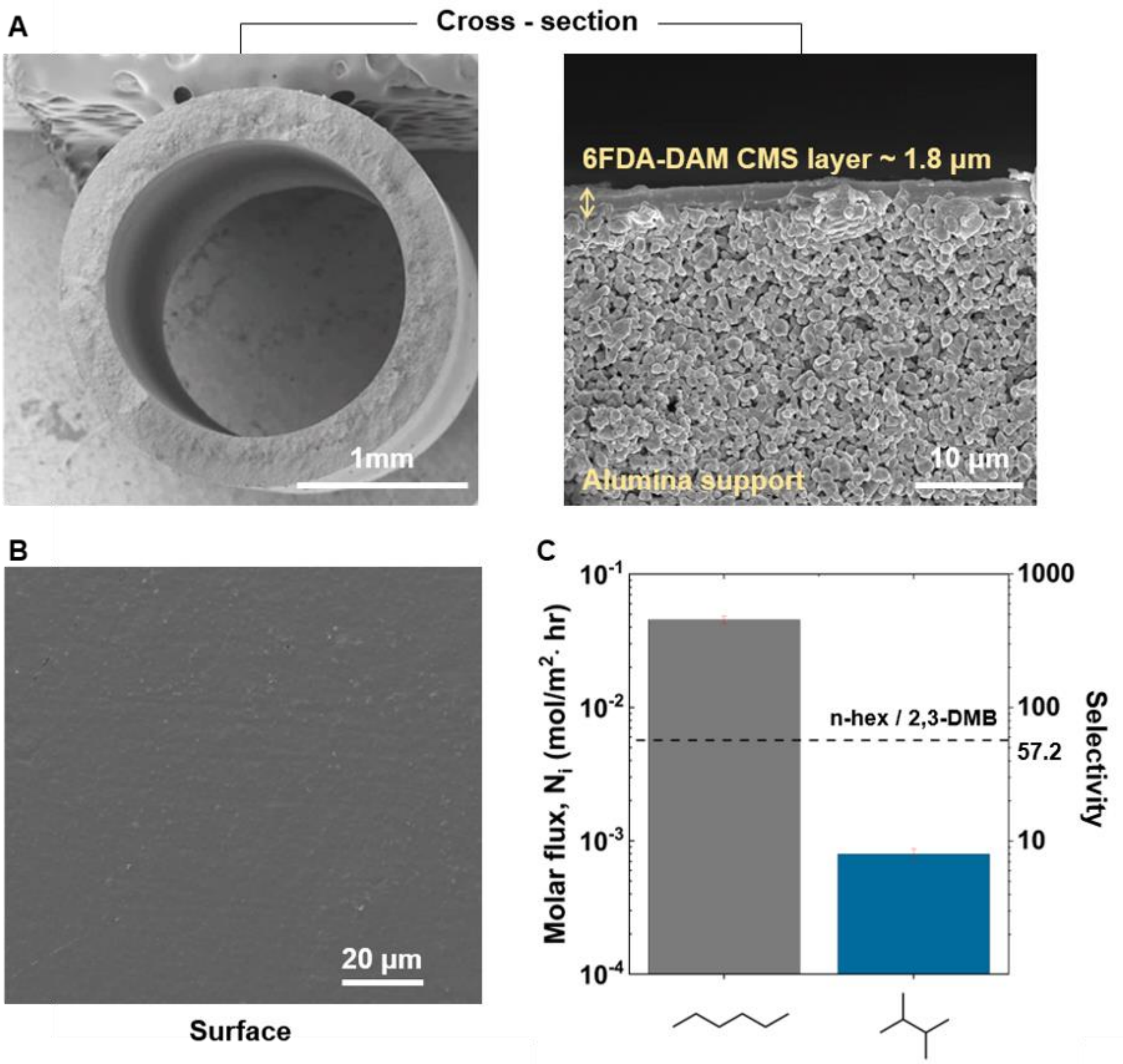


**Figure S28. (A)** Cross-sectional and **(B)** surface SEM images of alumina supported 6FDA − DAM CMS utilized in single OSFO test, **(C)** Single component OSFO experiment with *n*-hex and 2,3-DMB as feed solvent

7

**Table S1.** Classification of organic solvent separation systems

| | **Process type** | **Driving force** | **Transmembrane pressure (bar)** | **MWCO ($g\ mol^{-1}$)** |
|---|---|---|---|---|
| Dialysis | Osmotically-driven | Chemical potential gradient (Concentration gradient) | ~ 0 | 600 – 50000 |
| OSN | Pressure-driven | Pressure gradient | 5 – 40 | 200 – 1000 |
| OSRO | Pressure-driven | Pressure gradient | > 40 | < 200 |
| OSFO | Osmotically-driven | Chemical potential gradient (Concentration gradient) | ~ 0 | < 200 |

**Table S2.** Molecular weight and polydispersity index (PDI) of polyimides

| Polyimide | *Mw* | PDI |
|---|---|---|
| 6FDA−DAM | 170000 | 3.92 |
| 6FDA−DAM:DABA(3:2) | 140000 | 3.61 |
| 6FDA:BPDA(1:1)−DAM | 140000 | 1.77 |

**Table S3.** Elemental fraction of precursor polyimides and CMS memrbranes pyrolyzed at 500°C

| | Weight fraction (%) | | | |
|---|---|---|---|---|
| | C | H | N | O |
| 6FDA−DAM | 58.71 | 2.84 | 4.64 | 12.86 |
| 6FDA−DAM:DABA(3:2) | 56.46 | 2.60 | 4.61 | 19.38 |
| 6FDA:BPDA(1:1)−DAM | 63.8 | 3.39 | 5.29 | 12.45 |
| 6FDA−DAM CMS | 69.12 | 2.78 | 5.48 | 10.64 |
| 6FDA−DAM:DABA(3:2) CMS | 69.23 | 3.22 | 5.72 | 6.64 |
| 6FDA:BPDA(1:1)−DAM CMS | 71.06 | 2.8 | 5.85 | 8.99 |

**Table S4.** Sorption coefficients and sorption selectivities of hexane isomers in each CMS at T=25°C

| | $S_i$ ($10^{-3}$ mmol $g^{-1}$ $mmHg^{-1}$) | | | Sorption Selectivity, $S_i/S_j$ | | |
|---|---|---|---|---|---|---|
| | $S_{n\text{-hex}}$ | $S_{2\text{-MP}}$ | $S_{2,3\text{-DMB}}$ | $\frac{S_{n-\mathrm{hex}}}{S_{2-\mathrm{MP}}}$ | $\frac{S_{n-\mathrm{hex}}}{S_{2,3-\mathrm{DMB}}}$ | $\frac{S_{2-\mathrm{MP}}}{S_{2,3-\mathrm{DMB}}}$ |
| 6FDA−DAM CMS | 18.73 | 12.25 | 7.96 | 1.53 | 2.36 | 1.54 |
| 6FDA−DAM:DABA(3:2) CMS | 18.81 | 13.29 | 10.85 | 1.41 | 1.73 | 1.22 |
| 6FDA:BPDA(1:1)−DAM CMS | 15.37 | 10.48 | 6.82 | 1.47 | 2.25 | 1.54 |

**Table S5.** Diffusion coefficients and shape selectivities of hexane isomers in each CMS at T=25°C

| | Diffusion selectivity, $D_{o,\,i}$ / $D_{0,\,j}$ | | | Shape selectivity | | |
|---|---|---|---|---|---|---|
| | $\frac{D_{0,\ n\text{−hex}}}{D_{0,\ 2\text{−MP}}}$ | $\frac{D_{0,\ n\text{−hex}}}{D_{0,\ 2,3\text{−DMB}}}$ | $\frac{D_{0,\ 2\text{−MP}}}{D_{0,\ 2,3\text{−DMB}}}$ | $\frac{n-\text{hex}}{2-\text{MP}}$ | $\frac{n-\text{hex}}{2,3-\text{DMB}}$ | $\frac{2-\text{MP}}{2,3-\text{DMB}}$ |
| 6FDA−DAM CMS | 2.67 | 68.9 | 25.8 | .[a)] | . | 25.66 |
| 6FDA−DAM:DABA(3:2) CMS | 1.80 | 54.9 | 30.5 | . | . | . |
| 6FDA:BPDA(1:1)−DAM CMS | 7.09 | 70.0 | 9.87 | . | . | 1.56 |

[a)] ‘ . ’ denotes no contribution on diffusion selectivity

**Table S6.** Composition of 6FDA−DAM dope solution and bore fluid, and spinning parameters used in this study

| | Dope composition (wt%) | Bore composition (wt%) | Spinning parameter | Value |
|---|---|---|---|---|
| 6FDA−DAM | 18 | | Dope and bore fluid flow rate | 150 / 90 ml hr$^{-1}$ |
| NMP | 50.5 | | Take-up rate | 25 m min$^{-1}$ |
| THF | 10 | NMP / $H_2O$ = 90 / 10 | Quench bath temperature | 50°C |
| EtOH | 15 | | Spinneret temperature | RT |
| $LiNO_3$ | 6.5 | | Air gap height | 15cm |

**Table S7.** Comparison of operating conditions, flux and selectivity of membrane materials

| Membrane [ref] | Operating temperature (°C) | Feed composition | Phase of feed | Phase of permeate | Flux (mol $m^{-2}$ $hr^{-1}$) | | | Selectivity | |
|---|---|---|---|---|---|---|---|---|---|
| | | | | | linear | mono-branched | di-branched | linear/ di-branched | mono-branched / di-branched |
| Our work | 22 | Equimolar ternary mixture [*n*-hex / 2-MP / 2,3-DMB] | Liquid | Liquid | $6.85\times10^{-4}$ | $2.13\times10^{-4}$ | $1.66\times10^{-5}$ | 41.3 | 12.8 |
| *Beta zeolite tubular membrane vapor separation [2] | 100 | Equimolar quaternary mixture [*n*-hex / 3-MP / 2,2-DMB / 2,3-DMB] | Vapor | Vapor | $2.06\times10^{-2}$ | $1.39\times10^{-2}$ | $1.25\times10^{-2}$ | 1.64 | 1.11 |
| | 100 | | Vapor | Vapor | $3.12\times10^{-2}$ | $2.55\times10^{-2}$ | $2.27\times10^{-2}$ | 1.38 | 1.13 |
| | 70 | | Vapor | Vapor | $2.85\times10^{-2}$ | $2.24\times10^{-2}$ | $1.96\times10^{-2}$ | 1.46 | 1.15 |
| | 100 | | Vapor | Vapor | $3.05\times10^{-2}$ | $2.35\times10^{-2}$ | $2.01\times10^{-2}$ | 1.52 | 1.17 |
| | 130 | | Vapor | Vapor | $3.25\times10^{-2}$ | $2.46\times10^{-2}$ | $2.13\times10^{-2}$ | 1.53 | 1.16 |
| | 170 | | Vapor | Vapor | $3.34\times10^{-2}$ | $2.59\times10^{-2}$ | $2.19\times10^{-2}$ | 1.53 | 1.18 |
| | 100 | | Vapor | Vapor | $3.10\times10^{-2}$ | $2.55\times10^{-2}$ | $2.33\times10^{-2}$ | 1.33 | 1.09 |
| | 100 | | Vapor | Vapor | $2.66\times10^{-2}$ | $2.48\times10^{-2}$ | $2.40\times10^{-2}$ | 1.11 | 1.03 |
| | 100 | | Vapor | Vapor | $3.38\times10^{-2}$ | $3.11\times10^{-2}$ | $2.94\times10^{-2}$ | 1.15 | 1.06 |
| H-ZSM-5 tubular membrane vapor separation [3] | 27 | equimolar linear / di-branched mixture | Vapor | Vapor | 3.93 | . | 0.39 | 10 | . |
| | 77 | | Vapor | Vapor | 16.9 | . | 0.71 | 24 | . |
| | 117 | | Vapor | Vapor | 107 | . | 3.07 | 35 | . |
| H-ZSM-5 tubular membrane pervaporation [3] | 27 | equimolar linear / di-branched mixture | Liquid | Vapor | 1.07 | . | $7.66\times10^{-3}$ | 140 | . |
| | 77 | | Liquid | Vapor | 2.88 | . | $4.29\times10^{-3}$ | 671 | . |
| | 117 | | Liquid | Vapor | 9.00 | . | $9.00\times10^{-3}$ | 1000 | . |

**Table S8.** Comparison of heat duty in the distillation column for direct separation of hexane isomers and TIPB recovery

| Feed composition (mol ratio) | Minimum Number of Plates | Heat duty of condenser (kJ $hr^{-1}$) | Heat duty of reboiler (kJ $hr^{-1}$) |
|---|---|---|---|
| *n*-hex : 2,3-DMB = 5 : 5 | 41.6 | $-9.45 \times 10^{6}$ | $1.03 \times 10^{7}$ |
| *n*-hex : TIPB = 1 : 9 | 3.48 | $-5.96 \times 10^{5}$ | $9.58 \times 10^{6}$ |
| 2,3-DMB : TIPB = 1 : 99 | 3.52 | $-5.18 \times 10^{4}$ | $9.82 \times 10^{6}$ |

**Supplementary Note**

**S1. Classification of membrane-based solvent separation**

Various membrane processes have been studied to recover low molecular-weight organic solvents from the complex solution. These technologies include organic solvent nanofiltration (OSN), organic solvent reverse osmosis (OSRO), dialysis, and organic solvent forward osmosis (OSFO), which are liquid-phase separation techniques with no phase change involved in the processes. These processes could be classified depending on their driving force, transport mechanism, and molecular specificity (i.e., molecular weight cut-off (MWCO)) (**Figure S1**).

Most solvent separation methods employ a transmembrane concentration gradient or (hydraulic) pressure gradient for the permeation of molecules across the membrane. The technique using concentration gradient is a so-called osmotically-driven process, which utilizes draw solution (or draw solvent like in this study) downstream without external pressurization. As illustrated in **Figure S1A**, permeation occurs along the concentration gradient from feed to permeate side, though transmembrane pressure is zero in an osmotically-driven process. The utilization of the ‘draw’ solution on the downstream side of the membrane maintains the concentration gradient of permeable species from upstream to downstream. The concentration gradient results in a chemical potential gradient toward the downstream, leading the osmotic pressure gradient of light component between the solution to be concentrated (feed) and the solution of higher osmotic pressure than the feed. The osmotic pressure gradient of the light component generated toward the upstream side results in the natural tendency of the light component to flow downstream. Thus, the draw solution enables the extraction of the upstream target molecules into the downstream without external hydraulic pressure. Both dialysis and OSFO processes follow an osmotically-driven transport

mechanism with no transmembrane pressure. The pressure-driven processes, OSN and OSRO, adopt pressurization on the feed side to overcome the osmotic pressure gradient. It enables the permeation of molecules opposite the forward osmosis route.

More in detail, those processes can be subdivided based on the mass transport mechanism (**Figure S1B**). The molecular transport pathway within OSFO and OSRO membrane have a very similar dimension to the size of target solvent molecules, resulting in solution-diffusion mass transport. On the other hand, the dialysis and OSN membranes possess a larger pathway than OSFO and OSRO membranes. In this case, permeate molecules can interact with each other away from the membranes and can be characterized by a combination of ‘solution-diffusion and ‘pore-flow transport’ mechanisms. Indeed, these differences are shown as their MWCO values. The dialysis and OSN show MWCO much above 200 g mol-1, appropriate for solvent recovery from the solute. The OSFO and OSRO show precise molecular specificity (i.e., MWCO < 200g mol-1) that enables solvent–solvent separation. Standard features of the solvent separation methods are described in **Table S1**[2-4].

Conventional FO systems in water purification applications utilize aqueous draw solutions. They have many advantages over the traditional RO systems, mainly associated with treating feeds with higher salinity. In the last few years, this concept has been applied to organic solvent separation, and OSFO has emerged as a new technique that can overcome the limitation of OSRO. It is challenging to use OSRO with lean mixtures due to the high osmotic pressure gradient (e.g., xylene isomer separation). The OSFO processes employ a molecule with a large kinetic diameter sufficient to be rejected by a membrane as the draw solution (1,3,5-triisopropyl benzene in this study) to extract the more permeable component from a feed mixture osmotically. Notably, previous OSFO membranes suffer from reverse salt flux and membrane fouling, although draw solute has been utilized, whereas little work has been

considered size- and shape-selective OSFO processes for the molecular separation.

## S2. Estimation of the pore size distribution of three different CMS Membranes

It is challenging to generate an exact atomistic model of the CMS membrane because it is generally amorphous. Therefore, we combined the atomistic molecular simulation and linear programming to estimate the pore size distribution of three different CMS membranes. In this work, we modeled the pores of the membrane by creating 10 graphite sheets with different pore spacings (from 3.9 angstroms up to 7.4 angstroms) between the sheets to mimic the various pore sizes of the CMS membranes (**Figure 1D**).

We carried out grand canonical Monte Carlo (GCMC) simulations for a range of pressure points (from 0.000000001 Pa up to ~$10^5$ Pa) to compute the nitrogen ($N_2$) and argon (Ar) isotherms at T = 77 K and 87 K, respectively. Each GCMC simulation in the nitrogen isotherms consisted of a total of 30,000 cycles where the first 10,000 cycles were for initialization, and the remaining 20,000 cycles were for the production run to compute the ensemble averages for nitrogen uptake. Each GCMC simulation in the argon isotherms consisted of a total of 100,000 cycles where the first 50,000 cycles were for initialization, and the remaining 50,000 cycles were for the production run to compute the ensemble averages for argon uptake. Monte Carlo (MC) moves for $N_2$ consisted of swaps (insertions and deletions), re-insertions, rotations and translations with a ratio of 4:2:1:1, while MC moves for Ar consisted of swaps, re-insertion and translation with a ratio of 4:2:1. The chemical potential necessary to impose the gas phase fugacity was calculated from the gas-phase temperature and pressure using the Peng-Robinson equation of state.

The adsorbate-adsorbate and adsorbate-framework interactions were approximated based on the Lennard-Jones 12-6 potential (Equation (S1)):

$$V_{ij}(r_{ij}) = 4\varepsilon_{ij}\left[\left(\frac{\sigma_{ij}}{r_{ij}}\right)^{12} - \left(\frac{\sigma_{ij}}{r_{ij}}\right)^{6}\right] \qquad \text{(S1)}$$

where $V_{ij}$ is the interaction energy between atom $i$ and $j$, $\varepsilon_{ij}$ is the potential well depth between atom $i$ and $j$, $\sigma_{ij}$ is the van der Waals (vdW) diameter between atom $i$ and $j$, $r_{ij}$ is the distance between atom $i$ and $j$. LJ parameters for the framework atoms are from the DREIDING force field [5], while the $N_2$ and Ar molecules were modeled based on the parameters from the TraPPE [6] force field and the work of Reid et al [7], respectively. The parameters for different atom type interactions were calculated with the Lorentz-Berthelot mixing rules (Equation (S2) and (S3):

$$\sigma_{ij} = \left(\sigma_{ii} + \sigma_{jj}\right)/2 \qquad \text{(S2)}$$

$$\varepsilon_{ij} = \sqrt{\varepsilon_{ii}\varepsilon_{jj}} \qquad \text{(S3)}$$

The non-bonded interactions were cut-off at 14.0 Å with the analytic tail correction for both adsorbate-adsorbate and adsorbate-adsorbent interactions. The periodic boundary conditions (PBC) were applied to all three dimensions to satisfy the minimum image conventions. Carbon atoms forming the graphite sheets were immobile during the GCMC simulations. All GCMC simulations were carried out with the open-source RASPA 2.0 software [8].

The BET surface areas were calculated based on the simulated $N_2$ and Ar adsorption isotherms using SESAMI 1.0 software [8], which automatically computes the BET area by satisfying the four consistency criteria suggested by Rouquerol and coworkers [9]. Structural features of graphite sheets, such as pore size distribution (PSD) and accessible pore volume, were computed using Zeo++ software [10]. The pore size distribution (PSD) of each graphite-sheet model was calculated using a rigid sphere with a radius of 1.655 Å (the LJ σ parameter of nitrogen in the TraPPE model) to obtain the pore diameter of each graphite-sheet model. The dominant peak from pore size distribution (PSD) was used for the pore diameter of each graphite-sheet model. The accessible pore volume of each graphite-sheet model was calculated with a radius of 0 Å.

The structural features computed from the molecular models were used with experimental data obtained from real CMS membrane samples to estimate the pore size distribution of the CMS membranes. For this purpose, we fit a continuous linear function to the structural feature data from the simulation (saturation loading, BET surface area, and pore volume as a function of pore diameter), so that the value of each pore diameter could be predicted as a function of structure features. The spacing between the pore diameters was varied between 0.2 and 0.5. angstroms, starting from 3.9 angstrom up to 7.5 angstrom.

Using the data, we computed the relative error between each estimated value and experimental data (pore volume, saturation loading and BET surface area of three CMS membranes). The sum of relative errors between the simulation and experiments were computed for each structural feature for different distributions and combinations of pore diameters. A linear programming approach was used to find the combination of pore diameters that minimizes the total sum of errors in the pore volume, saturation loading and BET surface area. The constraints used in linear programming were that the values of each structural feature predicted according to the pore size distribution obtained had an error of less than 6 ~ 8 % from the experimental values. Code, data, and analysis scripts to reproduce the data are available in: https://gist.github.com/yoonseonghyun/c6ca4e78b38545865fb03fb229cd2515

## S3. Mass transport in CMS membrane

For He/$N_2$ selectivity measurement, the mass flux across the membrane is characterized by permeability, $P_A$, which is equal to the product of flux ($n_A$) and thickness ($l$) normalized by the transmembrane pressure gradient ($\Delta p_A$) as in Equation (S4). On the other hand, the skin layer thickness of asymmetric hollow fiber membranes is hard to determine, and its productivity is characterized by permeance rather than permeability, which is given as thickness normalized permeability (Equation (S5)). The efficiency of separation performance is explained in terms of (perm)selectivity defined as the ratio of permeability of a faster penetrant over a slower one. (Equation (S6))

$$P_A = \frac{[n_A][l]}{[\Delta p_A]},\ \text{1barrer} = 10^{-10}\ \text{cm}^3\ \text{[STP] cm cm}^{-2}\ \text{s}^{-1}\ \text{cmHg}^{-1})$$ Equation (S4)

$$\frac{P_A}{[l]} = \frac{[n_A]}{[\Delta p_A]},\ \text{1GPU} = 10^{-6}\ \text{cm}^3\ \text{[STP] cm}^{-2}\ \text{s}^{-1}\ \text{cmHg}^{-1}$$ Equation (S5)

$$\alpha_{A/B} = \frac{P_A}{P_B}$$ Equation (S6)

The mass transport in the CMS obeys the sorption–diffusion mechanism [11], therefore, the permeability and selectivity can be expressed with diffusion coefficient ($D_A$) and sorption coefficient ($S_A$). (Equation (S7))

$$P_A = D_A \times S_A,\ \alpha_{A/B} = \frac{D_A}{D_B} \times \frac{S_A}{S_B}$$ Equation (S7)

The permselectivity, divided into diffusion selectivity and sorption selectivity, could be further decoupled by employing the Eyring theory of rate processes with negligible volume change in the diffusion [12], in terms of enthalpic selectivity and entropic selectivity (Equation (S8)),

$$\frac{D_A}{D_B} = [exp(-\frac{E_{D,A} - E_{D,B}}{RT})]\ [exp(\frac{S_{D,A} - S_{D,B}}{R})]$$ Equation (S8)

where $R$, $T$, $E_D$, $S_D$ are the universal gas constant, temperature, activation energy of diffusion, and activation entropy of diffusion, respectively. In particular, the entropic contribution term, $exp\,(\frac{S_{D,A}-S_{D,B}}{R})$, is a function of the degree of freedom, which depends primarily on the molecular shape in addition to molecular size (Equation (S9)). $F'$ is the degree of freedom at the transition state of molecules localized around ultramicropores with diffusional activation energy, while F is the degree of freedom at a normal state. Such difference in activation entropy of diffusion arises from the rigid pore structure of the CMS, which imposes a restriction on the degree of freedom for molecules in the activated diffusion state in the ultramicropores.

$$exp(\frac{S_{D,A}-S_{D,B}}{R}) = \frac{(\frac{F'}{F})_A}{(\frac{F'}{F})_B} \qquad \text{Equation (S9)}$$

The gravimetric vapor sorption isotherms were utilized directly to determine the sorption behavior of hexane isomers in the CMS membranes (**Figure S14-16**). Sorption coefficients were calculated by dividing amounts of sorption by absolute pressure at $p/p_0$ = 0.85.

The Fickian diffusion coefficient ($D_i$ cm$^2$ s$^{-1}$) was determined using the appropriate Fickian diffusion model for this system [13] (flat sheet membrane geometry composed of non-swelling material) as given in Equation (S10). An exponential boundary condition, $\beta$, was employed since the relative pressure in the sample chamber does not vary instantaneously. The time-dependent uptake profiles were fitted using MATLAB to obtain $D_i$.

$$\frac{M_t}{M_\infty} = 1 - exp(-\beta t)\left(\frac{D}{\beta l^2}\right)^{\frac{1}{2}} tan\left(\frac{\beta l^2}{D}\right)^{\frac{1}{2}} - \frac{8}{\pi^2}\sum_{n=0}^{\infty}\frac{exp\{-(2n+1)^2\pi^2\cdot\frac{Dt}{4l^2}\}}{(2n+1)^2[1-(2n+1)^2\left(\frac{D\pi^2}{4\beta l^2}\right)]} \qquad \text{Equation (S10)}$$

As-calculated Fickian diffusion coefficients include concentration dependence of penetrant

activity, and thus, Darken's equation was employed to compensate for such dependence (Equation (S11)), where $q_i$ and $p_i$ are the loading amounts and activity of hexane isomers, relatively.

$$D_{0,i} = D_i \frac{d\ lnq_i}{d\ lnp_i} \qquad \text{Equation (S11)}$$

The activation energy of diffusion was calculated with the thermodynamically-corrected diffusion coefficients ($D_{0,\,i}$, cm$^2$ s$^{-1}$) at 25, 35, and 45 °C, based on the logarithmic expression of an Arrhenius relationship (Equation (S12)), where the $R$, $T$, $Đ_{0,i}$ are the universal gas constant, absolute pressure, and pre-exponential factor, respectively.

$$lnD_{0,i} = -\frac{E_{d,i}}{R}\frac{1}{T} + ln(Đ_{0,i}) \qquad \text{Equation (S12)}$$

**S4. Organic solvent forward osmosis**

Based on the solution-diffusion model [11], the overall driving force resulting in permeation of chemical species $i$ is the chemical potential gradient that could be interrelated with the concentration and pressure (Equation (S13))

$$d\mu_i = RT\ dln(\gamma_i x_i) + v_i dp \qquad \text{Equation (S13)}$$

where $\mu_i$, $\gamma_i$,, $x_i$,,$\upsilon_i$ and $p$ are the chemical potential, activity coefficient, mol fraction, molar volume of chemical species $i$, and pressure, respectively. For incompressible phases including liquid (feed solution, draw solvent) and solid membrane material, the molar volume of species i is independent of pressure, and as a result, integrating Equation (S13) yields

$$\mu_i = \mu_i{}^o +\ RT\ ln(\gamma_i x_i) + v_i(p - p_i{}^{sat}) \qquad \text{Equation (S14)}$$

where $p_i{}^{sat}$ is a saturation vapor pressure of i and $\mu_i{}^o$ is chemical potential of i at a $p_i{}^{sat}$.

In the OSFO process, the pressure is the same on both sides (i.e., feed side (shell side) and draw side (bore side) in this study) of the membrane (**Figure S26A**). Assuming osmotic equilibrium across the membrane can lead to a mathematical description of osmotic pressure as a function of activity and molar volume. As illustrated in **Figure S26B**, pressure applied on the draw side leads to osmotic equilibrium (net flow across the membrane is zero) and the pressure within the membrane is assumed to be equal to the high pressure ($p_l$). There exists discontinuity in pressure and activity ($\gamma_i x_i$), where the pressure difference $(p_l - p_O)$ is equal to osmotic pressure gradient, $\Delta\pi$. Applying Equation (S14) on both sides of the feed interface gives

$$\mu_{iO} = \mu_i{}^o +\ RT\ ln(\gamma_{iO} x_{iO}) + v_i(p_O - p_i{}^{sat}) \qquad \text{Equation (S15)}$$

$$\mu_{iO}{}^m = \mu_i{}^o +\ RT\ ln(\gamma_{iO}{}^m x_{iO}{}^m) + v_i(p_l - p_i{}^{sat}) \qquad \text{Equation (S16)}$$

where $X_i{}^m$ denotes physical properties of i in the membrane phase.

Since $\mu_{io} = \mu_{io}{}^{m}$ and $\gamma_{io}{}^{m} x_{io}{}^{m} = \gamma_{il} x_{il}$,

$$RT\, ln(\gamma_{io} x_{io}) + v_i p_o = RT\, ln(\gamma_{il} x_{il}) + v_i p_l \qquad \text{Equation (S17)}$$

$$\Delta\pi = p_l - p_o = \frac{RT}{v_i}\, ln(\frac{\gamma_{io} x_{io}}{\gamma_{il} x_{il}}) \qquad \text{Equation (S18)}$$

In summary, the OSFO process is driven by the osmotic pressure gradient (Equation (18)) without any external pressurization (**Figure S25**).

To calculate osmotic pressure gradient of each isomer across the membrane, molar volume, activity coefficient and concentration (i.e. mol fraction) must be specified. Molar volume of hexane isomers is already known value and mol fraction can be obtained from GC analysis, while the activity coefficients should be estimated. The activity coefficients of hexane isomers in mixture phase were estimated based on NRTL model. During the OSFO experiments, the volume of total feed mixture and flow rate of draw solvent is further higher compared to permeance values of hexane isomers, whereas permeance of hexane isomers was maintained almost constant. Therefore, we assumed constant concentration gradient of hexane isomers across the membrane, where the concentration of each isomer right after the membrane wall might be equal to the concentration at collected permeate.

For example,

- Feed solution: equimolar ternary mixture
- *n*-hex mol fraction in permeate = $6.05 \times 10^{-4}$
- 2-MP mol fraction in permeate = $1.18 \times 10^{-4}$
- 2,3-DMB mol fraction in permeate = $2.02 \times 10^{-5}$
- RT value: ($8.31 \times 10^{-2}$ L bar $K^{-1}$ $mol^{-1}$) × (298.15K) = 24.78 L bar $mol^{-1}$

- Specific molar volume of *n*-hexane: 129.884 $cm^3$ $mol^{-1}$
- Specific molar volume of 2-MP: 131.113 $cm^3$ $mol^{-1}$
- Specific molar volume of 2,3-DMB: 129.544 $cm^3$ $mol^{-1}$
- Active surface area of CMS hollow fibers in module: 9.73 $cm^2$

The osmotic pressure gradient of *n*-hexane

$$\Delta\pi(n\text{-hex}) = \frac{24.78\ \mathrm{L\ bar\ mol^{-1}}}{129.884\ \mathrm{cm^3\ mol^{-1}} \times 10^{-3}\mathrm{L\ cm^{-3}}} \times \ln\left(\frac{0.9912948 \times 0.33}{0.91889 \times 6.05 \times 10^{-4}}\right) = 1217\ \mathrm{bar}$$

The osmotic pressure gradient of 2-MP

$$\Delta\pi(2\text{-MP}) = \frac{24.78\ \mathrm{L\ bar\ mol^{-1}}}{131.113\ \mathrm{cm^3 mol^{-1}} \times 10^{-3}\mathrm{L\ cm^{-3}}} \times \ln\left(\frac{1.00398 \times 0.33}{0.91727 \times 1.18 \times 10^{-4}}\right) = 1517\ \mathrm{bar}$$

The osmotic pressure gradient of 2,3-DMB

$$\Delta\pi(2,3\text{-DMB}) = \frac{24.78\ \mathrm{L\ bar\ mol^{-1}}}{129.544\ \mathrm{cm^3 mol^{-1}} \times 10^{-3}\mathrm{L\ cm^{-3}}} \times \ln\left(\frac{0.98537 \times 0.33}{0.91566 \times 2.02 \times 10^{-5}}\right) = 1870\ \mathrm{bar}$$

**Reference**


[1] C. A. Schneider, W. S. Rasband, K. W. Eliceiri, *Nat. Methods* **2012**, *9*, 671.

[2] A. K. Pabby, S. S. H. Rizvi, A. M. S. Requena, *Handbook of Membrane Separations: Chemical, Pharmaceutical, Food, and Biotechnological Applications*, *Second Edition*, CRC Press, **2015**.

[3] P. Marchetti, M. F. Jimenez Solomon, G. Szekely, A. G. Livingston, *Chem. Rev.* **2014**, *114*, 10735.

[4] R. P. Lively, D. S. Sholl, *Nat. Mater.* **2017**.

[5] S. L. Mayo, B. D. Olafson, W. A. Goddard, *J. Phys. Chem.* **1990**, *94,* 8897.

[6] J. J. Potoff, J. I. Siepmann, *AIChE J.* **2001**.

[7] R. C. Reid, J. M. Prausnitz, B. E. Poling, *The Properties of Gases and Liquids*, McGraw Hill Book Co., New York, **1987**.

[8] D. Dubbeldam, S. Calero, D. E. Ellis, R. Q. Snurr, *Mol. Simul.* **2016**, *42,* 81.

[9] J. Rouquerol, P. Llewellyn, F. Rouquerol, *Stud. Surf. Sci. Catal.* **2007**, *160,* 1016.

[10] T. F. Willems, C. H. Rycroft, M. Kazi, J. C. Meza, M. Haranczyk, *Microporous Mesoporous Mater.* **2012**, *149,* 134.

[11] J. G. Wijmans, R. W. Baker, *J. Memb. Sci.* **1995**, *107,* 1.

[12] S. Glasstone, K. J. Laidler, H. Eyring, *The Theory of Rate Processes*, McGraw-hill, **1941**.

[13] J. Crank, E. P. J. Crank, *The Mathematics of Diffusion*, Clarendon Press, **1979**.
